# Scanning gradiometry with a single spin quantum magnetometer

**DOI:** 10.1038/s41467-022-31454-6

**Published:** 2022-06-29

**Authors:** W. S. Huxter, M. L. Palm, M. L. Davis, P. Welter, C.-H. Lambert, M. Trassin, C. L. Degen

**Affiliations:** 1 Department of Physics, ETH Zurich, Otto Stern Weg 1, 8093 Zurich, Switzerland; 2 Department of Materials, ETH Zurich, Hönggerbergring 64, 8093 Zurich, Switzerland; 3 Department of Materials, ETH Zurich, Vladimir Prelog Weg 1-5/10, 8093 Zurich, Switzerland; 4 Quantum Center, ETH Zurich, 8093 Zurich, Switzerland

**Keywords:** Magnetic properties and materials, Scanning probe microscopy, Quantum metrology

## Abstract

Quantum sensors based on spin defects in diamond have recently enabled detailed imaging of nanoscale magnetic patterns, such as chiral spin textures, two-dimensional ferromagnets, or superconducting vortices, based on a measurement of the static magnetic stray field. Here, we demonstrate a gradiometry technique that significantly enhances the measurement sensitivity of such static fields, leading to new opportunities in the imaging of weakly magnetic systems. Our method relies on the mechanical oscillation of a single nitrogen-vacancy center at the tip of a scanning diamond probe, which up-converts the local spatial gradients into ac magnetic fields enabling the use of sensitive ac quantum protocols. We show that gradiometry provides important advantages over static field imaging: (i) an order-of-magnitude better sensitivity, (ii) a more localized and sharper image, and (iii) a strong suppression of field drifts. We demonstrate the capabilities of gradiometry by imaging the nanotesla fields appearing above topographic defects and atomic steps in an antiferromagnet, direct currents in a graphene device, and para- and diamagnetic metals.

## Abstract (web summary)

Scanning NV center magnetometry enables imaging of weak magnetic fields at the nanoscale. Huxter et al. achieve an order-of-magnitude improvement in sensitivity by converting a spatial field gradient into an AC field by mechanical oscillations of the sensor, and image stray fields from atomic steps in an antiferromagnet.

## Introduction

Nanoscale magnetic stray fields appearing at surfaces and interfaces of magnetically ordered materials provide important insight into the local spin structure. Such stray fields are present, for example, above magnetic domains and domain walls^1^, magnetic vortices^2^, spin spirals^3^, skyrmions^4^, or topographic steps and defects^5^, and often accompany other types of ordering, like ferroelectricity^6^. Similar stray fields also appear near flowing currents^7,8^ or materials with a magnetic susceptibility^9^. Therefore, stray field measurements are a general and versatile tool to study local material or device properties.

While the magnetic imaging of ferromagnetic and ferrimagnetic textures is well-established^10–12^, detection of the much weaker stray fields of, for example, antiferromagnets, multiferroics or nanoscale current distribution is a relatively new development. Quantum magnetometers based on single nitrogen-vacancy (NV) centers have recently led to exciting advances in this direction^13–19^. In their standard configuration, NV magnetometers image stray fields by scanning a sharp diamond probe over the sample surface and monitoring the static shift of the NV spin resonance frequency^20,21^. State-of-the-art scanning NV magnetometers reach a sensitivity to static fields of a few $\,\mu {{{{{{{\rm{T}}}}}}}}/\sqrt{{{{{{{{\rm{Hz}}}}}}}}}$^16,22^. This sensitivity is sufficient for imaging the domain structure of monolayer ferromagnets^22–24^ and uncompensated antiferromagnets^14–17^, however, it remains challenging to detect the even weaker stray fields of isolated magnetic defects, spin chains, or ideally compensated antiferromagnets. While higher sensitivities, on the order of $50\,{{{{{{{\rm{nT}}}}}}}}/\sqrt{{{{{{{{\rm{Hz}}}}}}}}}$, have been demonstrated using dynamic (ac) detection of fields^25,26^, this approach is limited to the few systems whose magnetization can be modulated, like isolated spins^27,28^.

Here, we demonstrate a gradiometry technique for highly sensitive imaging of static magnetization patterns. Our method relies on up-conversion of the local spatial gradient into a time-varying magnetic field using mechanical oscillation of the sensor combined with sensitive ac quantum detection. This operating principle is well known from dynamic force microscopy^29,30^ and has recently been combined with scanning superconducting quantum interference devices^31–33^. While mechanical oscillation has been explored with NV centers in various forms^21,34–38^, it has not been realized for sensitive imaging of general two-dimensional magnetic samples. As a striking demonstration, we show that scanning gradiometry is able to resolve the nanotesla magnetic stray fields appearing above single atomic terraces in antiferromagnetic Cr_2_O_3_. Imaging of nanoscale current patterns, magnetic susceptibility in metals, and reconstruction of field maps from gradiometry data is also demonstrated.

## Results

### Gradiometry technique

**Fig. 1:**
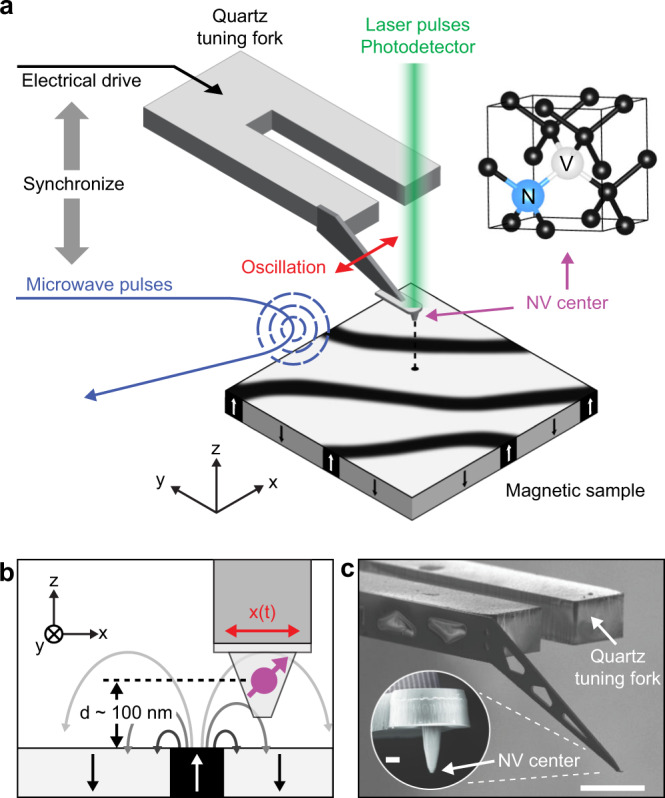
Experimental set-up. **a** Schematic of the scanning gradiometer. A single NV center located at the apex of a diamond tip is oscillated in shear-mode using a quartz tuning fork. The microwave pulse generation is synchronized, via a lock-in controller, with the electrical drive of the tuning fork. An objective located above the NV is used to apply laser pulses and collect the NV photo-luminescence. A three-axis piezo stage is used to position and scans the sample surface under the sensor. **b** Detail showing the orientations of sample, tip, NV center (purple), and direction of oscillation *x*(*t*). **c** Scanning electron micrograph of a quartz tuning fork and diamond tip (inset). Scale bars, 200 μm and 1 μm (inset).

The operating principle of our gradiometry technique is shown in Fig. 1a. Our scanning magnetometer set-up consists of a sample plate that is scanned underneath a diamond probe containing a single NV center at the tip apex formed by ion implantation^39^. The diamond tip is attached to the prong of a quartz tuning fork (Fig. 1) providing atomic force microscopy (AFM) position feedback^40^. The microscope apparatus additionally includes an objective to optically polarize and readout the NV center spin state and a bond wire acting as a microwave antenna for spin manipulation (see Methods for details). In the conventional mode of operation, we record a spin resonance spectrum at each pixel location to build up a map of the sample’s static stray field^16^.

To implement the gradiometer, we mechanically oscillate the NV in a plane parallel to the sample (shear-mode) by electrically driving the tuning fork at a fixed amplitude *x*_osc_ ~ 10–70 nm. The NV center now experiences a time-dependent field given by

1 $$B(x(t))= \,	B({x}_{0})+{\left.\frac{\partial B}{\partial x}\right|}_{x = {x}_{0}}{x}_{{{{{{{{\rm{osc}}}}}}}}}\sin (2\pi {f}_{{{{{{{{\rm{TF}}}}}}}}}t)\\ 	+{\left.\frac{{\partial }^{2}B}{\partial {x}^{2}}\right|}_{x = {x}_{0}}\frac{{x}_{{{{{{{{\rm{osc}}}}}}}}}^{2}}{2}{\sin }^{2}(2\pi {f}_{{{{{{{{\rm{TF}}}}}}}}}t)+\ldots$$

where *B* is the vector component of the sample’s stray field along the NV anisotropy axis (Methods), and where $x(t)={x}_{0}+{x}_{{{{{{{{\rm{osc}}}}}}}}}\sin (2\pi {f}_{{{{{{{{\rm{TF}}}}}}}}}t)$ describes the mechanical oscillation around the center location *x*_0_ with frequency *f*_TF_. The amplitudes of the 0*f*_TF_, 1*f*_TF_, and 2*f*_TF_ harmonics in leading orders of *x*_osc_ are given by

2a $${B}_{0}=B({x}_{0})$$

2b $${B}_{1}={x}_{{{{{{{{\rm{osc}}}}}}}}}{\left.\frac{\partial B}{\partial x}\right|}_{x = {x}_{0}}$$

2c $${B}_{2}=\frac{1}{2}{x}_{{{{{{{{\rm{osc}}}}}}}}}^{2}{\left.\frac{{\partial }^{2}B}{\partial {x}^{2}}\right|}_{x = {x}_{0}}$$

and are therefore proportional to the static field, gradient, and second derivative, respectively. The series expansion in Eq. (2) is accurate for oscillation amplitudes *x*_osc_ smaller than the scan height, typically *d* ~ 100 nm. Since the scan height sets the achievable spatial resolution^7^, the oscillation does not impair imaging resolution.

**Fig. 2:**
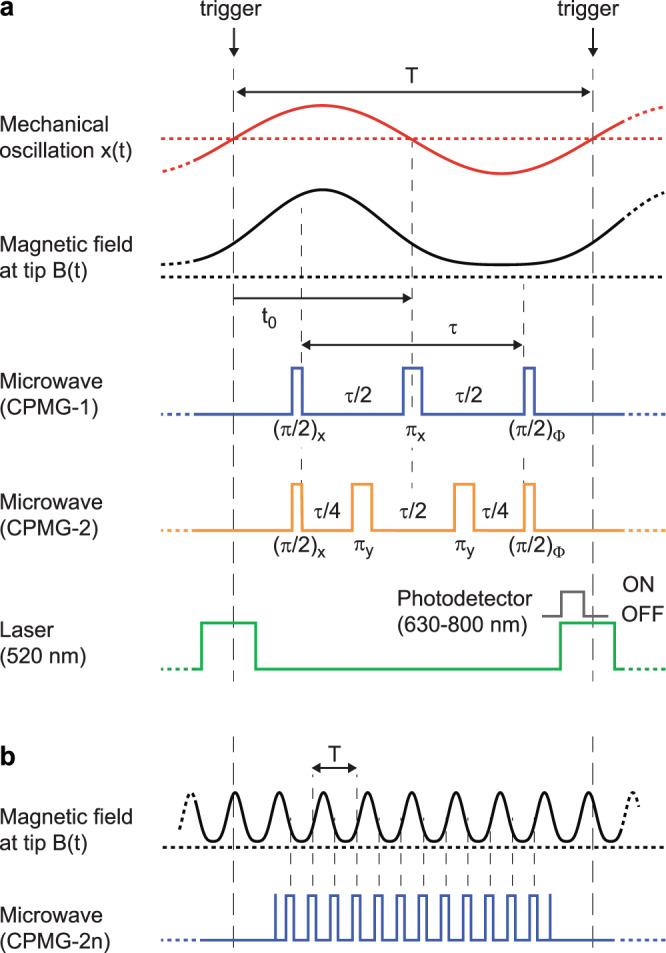
AC quantum sensing protocol for gradient detection. **a** Timing diagram of sensor oscillation (red), resulting in magnetic field at the sensor location (black), microwave pulse sequences for first and second harmonic (blue and orange), laser pulses (green), and photodetector collection window (gray). Pulse indicators such as (*π*)_x_ denote (angle)_axis_ of the spin rotation. *T* = 1/*f*_TF_ is the oscillation period, *τ* < *T* is the sensor interaction time, and *t*_0_ the trigger delay. We use a tuning fork with *f*_TF_ ~ 32 kHz. Durations of pulses are ~2 μs (laser) and ~100 ns (microwave *π*-rotation). **b** Scheme for detecting the first harmonic signal over *n* oscillation periods in the regime *τ* ≫ *T*. Here, *τ* = *nT* and the number of *π*-pulses is 2*n*.

To detect the harmonics of *B*(*t*), we synchronize the mechanical oscillation with a suitable ac quantum sensing sequence^41,42^, shown in Fig. 2a. Quantum sensing sequences measure the quantum phase accumulated by the coherent precession of a superposition of spin states during an interaction time *τ*^43^. To measure the *nf*_TF_ harmonic, we invert the spin precession *n* times during one mechanical oscillation period using microwave *π*-pulses (Carr-Purcell-Meiboom-Gill (CPMG-*n*) sequence^43,44^). The pulse protocols for the first (CPMG-1) and second (CPMG-2) signal harmonics are shown in Fig. 2a. The quantum phase accumulated for the first harmonic is given by

3 $$\phi 	= \int\nolimits_{t = {t}_{0}-\tau /2}^{{t}_{0}}{\gamma }_{{{{{{{{\rm{e}}}}}}}}}B(t){{{{{{{\rm{d}}}}}}}}t-\int\nolimits_{t = {t}_{0}}^{{t}_{0}+\tau /2}{\gamma }_{{{{{{{{\rm{e}}}}}}}}}B(t){{{{{{{\rm{d}}}}}}}}t\\ 	= -{\gamma }_{{{{{{{{\rm{e}}}}}}}}}{B}_{1}\,\frac{2{\sin }^{2}(\pi {f}_{{{{{{{{\rm{TF}}}}}}}}}\tau /2)}{\pi {f}_{{{{{{{{\rm{TF}}}}}}}}}}$$

where *γ*_e_ = 2*π* × 28 GHz/T is the gyromagnetic ratio of the NV electronic spin and *t*_0_ = *T*/2 (see Fig. 2a). The derivation of Eq. (3) and general expressions for higher harmonics and for sequences that are off-centered with respect to the tuning fork oscillation (*t*_0_ ≠ *T*/2) are given in Supplementary Note 1. To determine *ϕ* experimentally, we measure the photo-luminescence (PL) intensity *C*_Φ_ as a function of the phase Φ = *x*, *y*, −*x*, −*y* of the last microwave *π*/2 pulse (Fig. 2a). From the four PL signals *C*_Φ_ we then extract the phase using the two-argument arc tangent (see Methods),

4 $${\phi }^{({{\mbox{measured}}})}={{\mbox{arctan}}}\,\left(\frac{{C}_{-y}-{C}_{y}}{{C}_{x}-{C}_{-x}}\right)\,.$$

This four-phase readout technique has the advantage that the phase can be retrieved over the full 2*π* range with uniform sensitivity^19,26^. From *ϕ*^(measured)^ and Eq. (3) we then compute the gradient field *B*_1_. Since we can only measure *ϕ* modulo 2*π*, a phase unwrapping step is necessary for large signals that exceed the range [−*π*; *π*)^26^. Additionally, the oscillation amplitude can be used as a control parameter (*x*_osc_ < *d*) to dynamically set the maximum gradient before phase wrapping occurs.

For NV centers with long coherence times (*T*_2_ ≫ *T*), the sensitivity can be improved further by accumulating phase over multiple oscillation periods using CPMG-2*n* sequences (Fig. 2b). The simplest way to meet the *T*_2_ ≫ *T* condition is to use a mechanical oscillator with a higher resonance frequency. Alternatively, as demonstrated in this work, a higher mechanical mode of the tuning fork can be employed^45^. High-frequency detection has the added advantage that the interval between *π*-pulses (given by 1/(2*f*_TF_)) becomes very short, which makes dynamical decoupling more efficient and in turn leads to improved sensitivity^42,46^. We demonstrate single and multi-period detection schemes with sensitivities of $\sim \!\!120\,{{{{{{{\rm{nT}}}}}}}}/\sqrt{{{{{{{{\rm{Hz}}}}}}}}}$ and $\sim\!\!100\,{{{{{{{\rm{nT}}}}}}}}/\sqrt{{{{{{{{\rm{Hz}}}}}}}}}$, respectively, that exceed our best dc sensitivities of $\sim\!\!1-2\,\mu {{{{{{{\rm{T}}}}}}}}/\sqrt{{{{{{{{\rm{Hz}}}}}}}}}$ by one order of magnitude (Supplementary Notes 2 and 3).

### Demonstration of scanning gradiometry

**Fig. 3:**
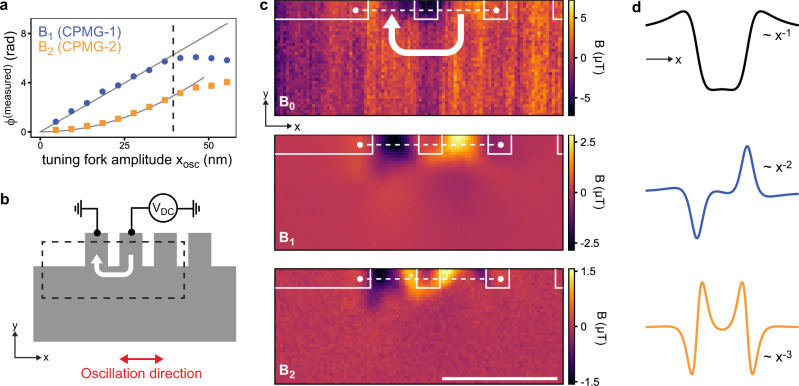
Two-dimensional scanning gradiometry of current flow in a graphene test geometry. **a** Measured and unwrapped quantum phase *ϕ*^(measured)^ plotted as a function of tip oscillation amplitude *x*_osc_ for the first (blue) and second (orange) harmonic. Solid lines are ∝ *x*_osc_ and $\propto {x}_{{{{{{{{\rm{osc}}}}}}}}}^{2}$, respectively. The dashed line indicates maximum oscillation amplitude before the Taylor approximation breaks down due to the onset of spatial averaging. **b** Schematic of the bilayer graphene device (gray). A direct current of *I*_dc_ ≈ 5.3 μA (white arrow) is applied between the indicated contacts (width ~400 nm). **c**, Static field map *B*_0_, gradient map *B*_1_, second derivative map *B*_2_ from the area indicated in **b**. Contours reflect the device layout. Dwell time is 30 s per pixel. Scale bar, 1 μm. **d**, Analytical spatial profiles for the *z* component of the magnetic field, gradient, and second derivative generated across the two contacts (dotted lines in **c**). The far-field decay with *x* is also indicated.

We begin measurements by calibrating the oscillation amplitude *x*_osc_ and verifying Eqs. (2, 3). We characterize the gradiometry phase detection by parking the tip over a fixed position on a magnetic test sample and measuring the *B*_1_ and *B*_2_ signals as a function of the oscillation amplitude *x*_osc_ (see Methods and Supplementary Note 4 for characterization and calibration details). Figure 3a confirms that the signals grow as *B*_1_ ∝ *x*_osc_ and ${B}_{2}\propto {x}_{{{{{{{{\rm{osc}}}}}}}}}^{2}$, as expected from the Taylor expansion (Eq. 2). Further, to avoid mixing of mechanical and field harmonic signals through surface interactions, we retract the tip by ~20 nm from the contact point while measuring (Supplementary Note 5).

We establish two-dimensional imaging by detecting the stray field from a direct current flowing in a bilayer graphene device (Fig. 3b). This device provides an ideal test geometry because the magnetic stray field and gradient can be tuned and directly compared to the analytical model. Moreover, results can be benchmarked against those obtained by ac current driving^26^. Figure 3c presents gradiometry maps *B*_1_ and *B*_2_ and, for comparison, a static field map *B*_0_ recorded using a standard dc technique^47^ with no tuning fork oscillation. Note that all images are recorded with the same imaging time and pixel size. The figure immediately highlights several advantages of gradiometry versus static field imaging: First, the signal-to-noise ratio is strongly enhanced due to the more sensitive ac detection, despite a lower absolute signal. Second, magnetic drift throughout the imaging time (several hours) is present in the static image but suppressed in the gradiometry images. Magnetic drifts are suppressed for two reasons; they fluctuate at low frequencies far away from the detection frequency, and magnetic drift sources do not generate appreciable magnetic field gradients over the oscillation range (~100 nm). Numerically differentiating the static image to remove field drifts is not suitable as it introduces high-frequency noise (Supplementary Fig. 6c). Third, because gradient fields decay quickly with distance (*B*_1_ ∝ *x*^−2^ and *B*_2_ ∝ *x*^−3^ compared to *B*_0_ ∝ *x*^−1^, see Fig. 3d and Supplementary Note 6), they offer a higher apparent image resolution and are thus easier to interpret.

### Imaging of antiferromagnetic surface texture

We now turn our attention to the imaging of stray fields above antiferromagnetic materials, focusing on the archetypical model system Cr_2_O_3_. Antiferromagnets represent a general class of weakly magnetic materials that are both challenging to the image by existing techniques^48–50^ and of key importance for understanding multiferroicity and topological magnetism in the context of antiferromagnetic spintronics^51^. Although antiferromagnets are nominally non-magnetic, weak stray fields can appear due to uncompensated moments at domain walls^14–16^, spin spirals^13^, topographic steps^17^, or surface roughness and defects. The latter is particularly difficult to detect, yet plays an important role in the pinning of domain walls^17^ and establishing an exchange bias at material interfaces^52^. Careful study of these weaker fields is therefore an important avenue for quantifying the interplay between surface roughness, stray field structure, local magnetization, and domain wall behavior.

**Fig. 4:**
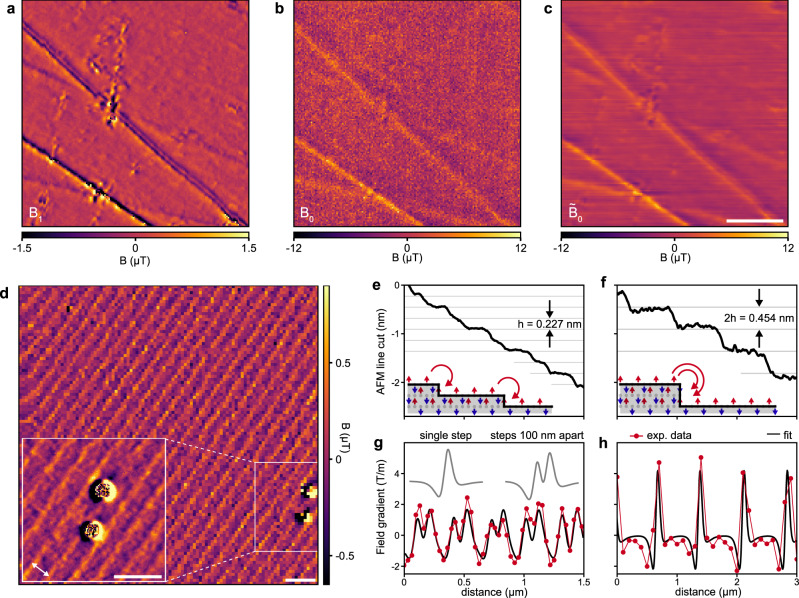
Topographic fields from antiferromagnetic Cr_2_O_3_. **a** Gradiometry map *B*_1_ of topographic defects from a polished Cr_2_O_3_ single crystal. Dwell time is 10 s per pixel and *x*_osc_ = 23 nm. **b** Corresponding static field map *B*_0_. Dwell time is 1 s per pixel. **c** Improved static field map ${\tilde{B}}_{0}$ obtained from a weighted average of **a** and **b** (see Methods). The noise standard deviation is reduced from 1.8 μT (**b**) to 90 nT in the oscillation direction (*x*-direction) and 340 nT in the *y*-direction (**c**). The directional sensitivity could be avoided using tapping mode oscillation (see Methods). When normalized by measurement time, *B*_1_ is more sensitive than *B*_0_ by a factor larger than 8. **d** *B*_1_ image of the stray field generated by atomic steps on an as-grown Cr_2_O_3_(0001) surface. Two magnetic defects which produce much larger gradient signals are also visible. Inset is a detail of defects and atomic steps recorded with higher pixel resolution. The distance between steps is ca. 400 nm (arrow). Dwell time is 5 s per pixel. Scale bars in **a**–**d** 1 μm. **e**, **f** AFM line scans revealing mono- and diatomic steps with different terrace sizes. *h* is the distance between neighboring O-planes along the Cr_2_O_3_*c*-axis^53,54^. Insets show sketches of atomic steps and up–down ordering of Cr^3+^ moments (red, blue). Curved arrows are field lines. **g**–**h** Gradiometry line scans acquired over different areas on the as-grown Cr_2_O_3_ surface. The profiles are fitted to a stray field model involving atomic steps (solid lines). *x*_osc_ = 46 nm in **d**, **g**, and **h**.

In Fig. 4a we show a gradiometry image of a polished Cr_2_O_3_ single-crystal^16^. Cr_2_O_3_ has a layered structure of out-of-plane Cr^3+^ moments at the (0001) surface^53,54^ that lead to stray fields at topographic steps^16,17^. These stray fields are proportional to the step height. Indeed, Fig. 4a reveals a rich variety of magnetic anomalies on this surface, including two ~5 nm deep and ~200 nm wide topographic trenches introduced by polishing, a number of point defects, and general texture of the ~2 nm-rms surface roughness (see Supplementary Fig. 11 for topographic characterization). A quantitative calculation of the expected stray field maxima shows that the magnetic anomalies are well explained by the surface topography (Supplementary Note 7). Except for the trenches, these surface defects are not visible in the static field image (Fig. 4b). Note that it is possible to convert the gradiometry map into an improved static field map through integration and weighted averaging in *k*-space (Fig. 4c and Methods). See Supplementary Fig. 6 for the reverse process, where a *B*_0_ image is converted into a *B*_1_ image.

Next, we show that gradiometry is sufficiently sensitive to detect single atomic surface steps of the layered Cr_2_O_3_(0001) surface. Figure 4d displays a gradiometry image from a second Cr_2_O_3_ single crystal with an as-grown (unpolished) surface^53^. We observe a striking pattern of regular stripes separated by a few hundred nanometers and an approximate amplitude of *B*_1_ ~ 250 nT. The pattern extends over the entire crystal surface with different stripe separations and directions in different regions of the sample (Supplementary Fig. 12). By converting *B*_1_ measurements into local gradients we deduce height changes <1 nm (Supplementary Note 7), suggesting that the repeating striped patterns are caused by single (*h* = 0.227 nm), or multiple atomic step edges. To corroborate, we correlate the magnetic features to the AFM topography of the sample (Supplementary Fig. 12). Figure 4e, f show line cuts perpendicular to the stripe direction in two regions of the sample surface, and reveal the presence of both mono- and diatomic step edges. The gradiometry data (Fig. 4g, h), are well fitted by a simple model (see Methods), producing a fitted surface magnetization of *σ*_*z*_ = 2.1 ± 0.5 μ_B_/nm^2^, in good agreement with earlier data^14,16^. Together, our findings unambiguously confirm the stepped growth of the Cr_2_O_3_(0001) surface^54^. To our knowledge, our work reports the first magnetic stray field imaging of atomic steps on an antiferromagnet. It opens a complementary path to existing atomic-scale techniques, such as spin-polarized scanning tunneling microscopy and magnetic exchange force microscopy^55^, without sharing the need for an ultra-high vacuum, a conducting surface, or cryogenic operation.

### Imaging of magnetic susceptibility

**Fig. 5:**
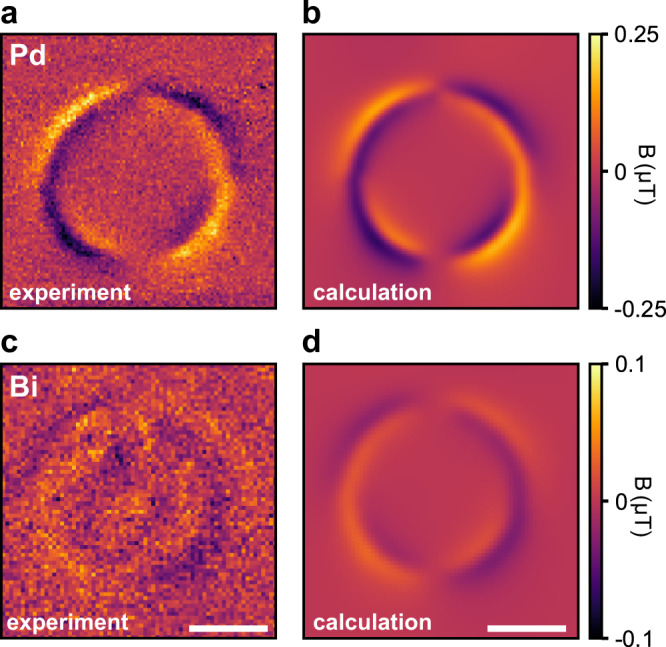
Nanoscale susceptometry. **a** Measured *B*_1_ image of a 2-μm-diameter paramagnetic Pd disc in a 35 mT bias field. **b** Calculated *B*_1_ image (best fit) of the Pd disc. **c**, **d** Corresponding measurement, and calculation for diamagnetic Bi in a bias field of 33 mT. Offsets of 51 nT and 38 nT due to the gradient of the external bias magnet are subtracted from **a** and **c**, respectively. See Methods for calculation and fit details. Scale bars, 1 μm.

We conclude our study by demonstrating nanoscale imaging of magnetic susceptibility. Susceptometry measurements are important for investigating, for example, the magnetic response of patterned metals and materials^9,56^, superconductors^57^ as well as para- and superparamagnetic nanoparticles^58,59^. Figure 5a shows a gradiometry map of a 50-nm-thick disc made from paramagnetic Pd placed in a bias field of *B*_ext_ = 35 mT. Under the bias field, the disc develops a magnetization of *M* = *χB*_ext_/μ_0_, where *χ*_Pd_ is the magnetic susceptibility of the Pd film. A fit to the data (see Methods), shown in Fig. 5b, produces a susceptibility of *χ*_Pd_ = (6.6 ± 0.2) × 10^−4^. This is slightly smaller than the value of pure Pd (*χ*_Pd_ = 7.66 × 10^−4^^60^). The decreased susceptibility may be attributed to either a finite-size effect^61^ or hydrogen adsorption^62^. Repeating the same experiment with Bi, a diamagnetic sample, produces an experimental value of *χ*_Bi_ = −(1.7 ± 0.1) × 10^−4^(Fig. 5c, d), which matches the accepted room temperature value of *χ*_Bi_ = − 1.67 × 10^−4^^60^. Despite the ~4 × weaker susceptibility, the magnetic pattern is clearly visible and its sign is inverted compared to the paramagnetic Pd disc. Additionally, the apparent local structure at the center of the disc is explained by a variation in the film thickness (Supplementary Fig. 13). Together, Fig. 4a–d demonstrate the feasibility of extending sensitive dc susceptometry to the nanometer scale.

## Discussion

Our work demonstrates a simple yet powerful method for imaging static magnetization patterns with high sensitivity and spatial resolution. While we demonstrate scanning gradiometry on a layered antiferromagnet, extension to more challenging systems including perfectly compensated collinear antiferromagnets, screw and step dislocations^5^, or isolated magnetic defects is natural. In particular, the magnetic signal from an atomic step edge is equivalent to that of a one-dimensional spin chain with a linear magnetization density of *σ*_*z*_*h* ≈ 0.5 μ_B_/nm (Methods), demonstrating the feasibility of imaging generic 1D spin systems. Gradiometry is also well-positioned for imaging the internal structure of domain walls and skyrmions, and in particular, for quantifying their size and chirality^16,63–65^ (Supplementary Fig. 14). Finally, while we demonstrate gradiometry on magnetic fields, the technique can be extended to electric fields by orienting the external bias magnetic field perpendicular to the NV axis^66–68^. In particular, the dynamic mode of operation may alleviate charge screening that has previously limited scanning NV electrometry of dc electric field sources^69^. With the ability to image both magnetic and electric fields, one could imagine correlating antiferromagnetic and ferroelectric order in multiferroics^51^, providing a unique angle to investigate the magneto-electric coupling in these fascinating and technologically important materials.

## Methods

### Experimental set-up

All experiments were carried out at room temperature with two home-built scanning microscopes. Micro-positioning was carried out by closed-loop three-axis piezo stages (Physik Instrumente) and AFM feedback control was carried out by a lock-in amplifier (HF2LI, Zurich Instruments) and standard PID controls. PL of the NV centers was measured with avalanche photodiodes (APDs) (Excelitas) and data were collected by standard data acquisition cards (PCIe-6353, National Instruments). Direct currents sent through the bilayer graphene were created with an arbitrary waveform generator (DN2.663-04, Spectrum Instrumentation) and the current was measured with a trans-impedance amplifier (HF2TA, Zurich Instruments). Microwave pulses and sequences were created with a signal generator (Quicksyn FSW-0020, National Instruments) and modulated with an IQ mixer (Marki) and an arbitrary waveform generator (DN2.663-04, Spectrum Instrumentation, and HDAWG, Zurich Instruments). NV centers were illuminated at < 100 μW by a custom-designed 520 nm pulsed diode laser. Scanning NV tips were purchased from QZabre AG^39^. Three different NV tips were used throughout this study with standoff distances *d* between 70 and 130 nm (excluding the 20-nm retract distance). When imaging our samples, we typically measured for 5 to 30 seconds per pixel to achieve good signal-to-noise. We observed relatively small spatial drifts (typically <30-nm per day). As a result, we did not employ any drift correction techniques and we do not expect drifts to affect our results or scientific claims.

### Magnetic samples

#### Bilayer graphene device

Standard microfabrication processes were used, including mechanical exfoliation and a dry transfer process for generating the hBN-bilayer-graphene-hBN stack, electron beam lithography, and plasma etching for creating the device geometry and physical vapor deposition for creating the metallic device contacts. Full details can be found in ref. ^26^.

#### Cr_2_O_3_ crystals

The mechanically polished and non-polished Cr_2_O_3_ bulk single crystals were provided by professor Manfred Fiebig. Both crystals have a (0001) surface orientation. Crystal growth and processing details can be found in refs. ^16,53^.

#### Pd and Bi discs

Metallic micro-discs were created using standard microfabrication processes. Discs were defined through electron beam lithography on spin-coated Si wafers. Chemical development of the spin-coated resist, followed by metal deposition of either 50-nm of Pd or Bi, and a lift-off process finished the fabrication.

### Initialization and readout of NV spin state

A laser pulse of ~2 μs duration was used to polarize the NV center into the *m*_*S*_ = 0 state. Then, the dc or ac quantum sensing measurement occurred on the *m*_*S*_ = 0 to *m*_*S*_ = −1 transition (see below). The readout of the NV’s spin state was performed by another ~2 μs long laser pulse, during which the photons emitted from the NV center were collected with the APD, binned as a function of time, and summed over a window (~300 ns) that optimized spin state-dependent PL contrast^70^.

### DC sensing protocol

DC magnetic images were acquired with the pulsed ODMR method^47^. Measured magnetic resonance spectra were fitted for the center frequency *f*_*c*_ of a Lorentzian function, $L(f)=1-\epsilon {[(2\pi f-2\pi {f}_{c})/{\omega }_{L}]}^{2}+1{\left.\right]}^{-1}$, where *ϵ* is the spin contrast (in percent) and *ω*_*L*_ is the width of the Lorentzian dip. In a 2D scan the magnetic field projected along the axis of the NV could be determined at each pixel using *B*(*x*, *y*) = 2*π*[*f*_*c*_(*x*, *y*) − *f*_0_]/*γ*_e_ where *f*_0_ is the resonance frequency far from the surface. For our diamond probes, the NV anisotropy axis was at an *θ* ~ 55^∘^ angle with respect to the out-of-plane direction (*z*-axis in Fig. 1). Therefore, *B*(*x*, *y*) corresponded to the vector field projection along this tilted direction.

### AC sensing protocol

AC sensing used either a spin echo or dynamical decoupling sequence^41,42^. The NV spin state was initialized optically into the *m*_*S*_ = 0 state followed by a microwave *π*/2-pulse to create a coherent superposition between the *m*_*S*_ = 0 and *m*_*S*_ = −1 states. The quantum phase *ϕ* accumulated between the two states during the coherent precession can be expressed as $\phi =\int\nolimits_{0}^{\tau }{\gamma }_{{{{{{{{\rm{e}}}}}}}}}g(t)B(t)dt$ where *g*(*t*) is the modulation function^43^, *B*(*t*) is the magnetic field, *τ* is the interaction time (the time in between the first and last *π*/2-pulse), and where we use the rotating frame approximation. The modulation function alternates between ± 1 with each microwave *π*-pulse during the pulse sequence. While imaging with the gradiometry pulse sequences, 3*π*/2-pulses were sometimes used instead of the final *π*/2-pulses for the projective spin readout to reduce pulse imperfections caused by (ca. ± 100 kHz) drifts in the NV resonance frequency.

### Calibration of tuning fork oscillation

We estimated the oscillation amplitude *x*_osc_ and oscillation angle in the *xy*-plane, denoted by *α*, of the tuning forks with two different in-situ measurement techniques. The first method involved processing a static field map and a gradient field map acquired over the same region with a least-squares minimization scheme. By minimizing a cost function proportional to the pixel value differences between the *B*_1_ and numerically differentiated *B*_0_ images, estimates for *x*_osc_ and *α* were determined. The second method involved stroboscopic imaging of the static field (*B*_0_) synchronized to the tuning fork oscillation. By measuring the time-tagged displacements of magnetic features recorded at different positions during the tuning fork oscillation, *x*_osc_ and *α* could be estimated by fitting to the path taken by the NV. We found that *x*_osc_ scaled linearly with the applied drive voltage for the voltages we used while imaging. Examples of these calibration methods are given in Supplementary Note 4.

### Calibration of the trigger delay

In order to optimize the ac sensing sequences, and to distinguish between signals from the first and second derivatives (see Supplementary Note 1), a trigger delay (*t*_0_) calibration measurement must be made. The calibration measurement consisted of measuring the phase *ϕ* at a stationary point on the sample, while varying *t*_0_, thus producing a phase that oscillated as a function of *t*_0_. The chosen value of *t*_0_ was the value that maximized the measured phase. Examples of this calibration measurement, as well as the characterization of PL oscillation caused by the tuning fork motion, are shown in Supplementary Note 4.

### Reconstruction of the static field map from the gradient map

A gradient map *B*_1_ can be used to reduce the noise and improve the image of a (less sensitive) static field map *B*_0_. While direct integration of *B*_1_ produces a *B*_0_ map^32^, a combination approach using *B*_0_ and *B*_1_ provides the best noise suppression across all spatial wavelengths, with no need for ad-hoc boundary conditions. Letting *B*_T_ denote the true static magnetic field value, the experimentally measured *B*_0_ and *B*_1_ field maps are

5 $${B}_{0}(x,y)	={B}_{{{{{{{{\rm{T}}}}}}}}}(x,y)+{w}_{0}\\ {B}_{1}(x,y)	={x}_{{{{{{{{\rm{osc}}}}}}}}}\frac{\partial }{\partial r}{B}_{{{{{{{{\rm{T}}}}}}}}}(x,y)+{w}_{1}$$

where $\frac{\partial }{\partial r}=\cos (\alpha )\frac{\partial }{\partial x}+\sin (\alpha )\frac{\partial }{\partial y}$ is the directional derivative and ${w}_{0} \sim {{{{{{{\mathcal{N}}}}}}}}(0,{\sigma }_{{B}_{0}}^{2})$ and ${w}_{1} \sim {{{{{{{\mathcal{N}}}}}}}}(0,{\sigma }_{{B}_{1}}^{2})$ are white noise added to each pixel (reflecting the Poissonian shot noise of the photo-detection). In *k*-space these equations can be transformed into

6 $${\hat{B}}_{0}({k}_{x},{k}_{y})	={\hat{B}}_{{{{{{{{\rm{T}}}}}}}}}({k}_{x},{k}_{y})+{\hat{w}}_{0}\\ \frac{{\hat{B}}_{1}({k}_{x},{k}_{y})}{i{x}_{{{{{{{{\rm{osc}}}}}}}}}{k}_{r}}	={\hat{B}}_{{{{{{{{\rm{T}}}}}}}}}({k}_{x},{k}_{y})+\frac{{\hat{w}}_{1}}{i{x}_{{{{{{{{\rm{osc}}}}}}}}}{k}_{r}}$$

where $\hat{X}$ denotes the Fourier transform of *X*, ${k}_{r}={k}_{x}\cos \alpha +{k}_{y}\sin \alpha$ is the dot product between the oscillation direction and the *k*-vector [*k*_*x*_, *k*_*y*_]. Integration in *k*-space introduces a *k*-dependent noise term in the gradient field map. In particular, the integrated gradient map has noise amplification near the line ${k}_{y}=-\cot (\alpha ){k}_{x}$ but has noise suppression far away from that line. Directional sensitivity could be avoided by oscillating the tuning fork in the *z*-direction (tapping mode), or by using an oscillator that supports orthogonal lateral modes. To circumvent this problem we average two *k*-space maps (Eq. (6)) with *k*-vector dependent weights that reflect the noise added by the integration process. The use of inverse variance weights additionally results in an image with the lowest possible variance. Thus, the optimal reconstructed static field map can be computed by taking the inverse Fourier transform of

7 $${\hat{\tilde{B}}}_{0}({k}_{x},{k}_{y})={\hat{B}}_{0}({k}_{x},{k}_{y})\,\frac{{k}_{0}^{2}}{{k}_{0}^{2}+{k}_{r}^{2}}+\frac{\hat{{B}}_{1}({k}_{x},{k}_{y})}{i{x}_{{{{{{{{\rm{osc}}}}}}}}}{k}_{r}}\,\frac{{k}_{r}^{2}}{{k}_{0}^{2}+{k}_{r}^{2}}$$

where ${k}_{0}^{2}={\sigma }_{{B}_{1}}^{2}/({x}_{{{{{{{{\rm{osc}}}}}}}}}^{2}{\sigma }_{{B}_{0}}^{2})$ defines a cut-off wave vector determined by the oscillation amplitude and the noise variances ${\sigma }_{{B}_{0}}^{2}$ and ${\sigma }_{{B}_{1}}^{2}$. The corresponding cut-off wavelength *λ* = 2*π*/*k*_0_ reflects the spatial wavelength above (below) which the *B*_0_ (*B*_1_) field map is less noisy. It should also be noted that the *k*-space averaging process can be modified to include the *B*_2_ map, however, the *B*_1_ map provides the most significant improvement. Specifically, in Fig. 4c, *x*_osc_ = 23 nm and *α* = 180^∘^ were used as reconstruction parameters (determined by the calibration in Supplementary Note 4). Note, since the gradient and *k*-space averaging are directional, the noise reduction is also directional (approximately 22 × in the *x*-direction and 5.6 × in the *y*-direction).

### Stray fields from atomic step edges

For an atomic step edge propagating along the *y*-direction the stray field is modeled by two out-of-plane magnetic samples with different heights. Taking the analytical form (see ref. ^63^) for the stray fields above an edge at *x* = 0 as ${B}_{x}(x,z)=\frac{-{\mu }_{0}{\sigma }_{z}}{2\pi }\frac{z}{{x}^{2}+{z}^{2}}$, *B*_*y*_ = 0, and ${B}_{z}(x,z)=\frac{{\mu }_{0}{\sigma }_{z}}{2\pi }\frac{x}{{x}^{2}+{z}^{2}}$, where *σ*_*z*_ is the surface magnetization, we define the stray field produced by a small change in the height *h* as

8 $$\begin{array}{ll}{B}_{x}^{{{{{{{{\rm{step}}}}}}}}}&={B}_{x}(x,d)-{B}_{x}(x,d+h)\approx \frac{{\mu }_{0}{\sigma }_{z}h}{2\pi }\frac{({x}^{2}-{d}^{2})}{{({x}^{2}+{d}^{2})}^{2}}\\ {B}_{z}^{{{{{{{{\rm{step}}}}}}}}}&={B}_{z}(x,d)-{B}_{z}(x,d+h)\approx \frac{{\mu }_{0}{\sigma }_{z}h}{2\pi }\frac{2xd}{{({x}^{2}+{d}^{2})}^{2}}\end{array}$$

with ${B}_{y}^{{{{{{{{\rm{step}}}}}}}}}=0$. The expressions are simplified in the limit of *h* ≪ *d* since the sub-nanometer atomic step edges are much smaller than typical standoff distances of *d* ~ 50 − 100 nm. We measure the gradient of the stray field along the oscillation direction, taken as the *x*-direction for simplicity. This leads to field gradients of

9 $$\frac{\partial {B}_{x}^{{{{{{{{\rm{step}}}}}}}}}}{\partial x}=\frac{{\mu }_{0}{\sigma }_{z}h}{\pi }\;\frac{x(3{d}^{2}-{x}^{2})}{{({x}^{2}+{d}^{2})}^{3}}\\ \frac{\partial {B}_{z}^{{{{{{{{\rm{step}}}}}}}}}}{\partial x}=\frac{{\mu }_{0}{\sigma }_{z}h}{\pi }\;\frac{d({d}^{2}-3{x}^{2})}{{({x}^{2}+{d}^{2})}^{3}}$$

The fits in Fig. 4g, h are produced by projecting the field gradients onto the NV axis defined by ${{{{{{{\bf{e}}}}}}}}=[\sin \theta \cos \varphi ,\sin \theta \sin \varphi ,\cos \theta ]$ and summing over multiple steps. The line cuts are produced by rotating and plane averaging a 2D image (Supplementary Fig. 12). We account for the rotation angle in the *xy*-plane by introducing an additional image rotation angle $\varphi ^{\prime}$. The experimentally measured *B*_1_ field is then fitted by

10 $$\frac{{B}_{1}}{{x}_{{{{{{{{\rm{osc}}}}}}}}}\cos (\varphi ^{\prime} )}=\mathop{\sum}\limits_{{{{{{{{\rm{steps}}}}}}}}}\sin (\theta )\cos (\varphi +\varphi ^{\prime} )\frac{\partial {B}_{x}^{{{{{{{{\rm{step}}}}}}}}}}{\partial x}+\cos (\theta )\frac{\partial {B}_{z}^{{{{{{{{\rm{step}}}}}}}}}}{\partial x}$$

We set *h* = 0.227 nm and $\varphi ^{\prime} =4{0}^{\circ }$ for the fit in Fig. 4g, *h* = 0.454 nm and $\varphi ^{\prime} =2{3}^{\circ }$ for the fit in Fig. 4h and fixed *x*_osc_ = 46 nm (determined by the tip calibration) in both fits. The standoff distance *d*, the surface magnetization *σ*_*z*_, angles *θ* and *φ* and step edge locations are free parameters in the fit. Collectively, the fitted line scans give *d* = 89 ± 12 nm (including the 20 nm retract distance) and *σ*_*z*_ = 2.1 ± 0.5 μ_B_/nm^2^, which is consistent with previous measurements on this sample^16^.

Note, Eq. (8) is functionally equivalent to the magnetic field from a one-dimensional ferromagnetic spin chain with a linear magnetization density of *M*^1*D*^ = *σ*_*z*_*h*. To demonstrate this, we calculate the stray field produced by a infinite line of magnetic dipoles along the line *x* = 0 and pointing in the *x*-direction as:

11 $${B}_{x}^{1{{{{{{{\rm{D}}}}}}}}}= \,\frac{{\mu }_{0}{M}^{1{{{{{{{\rm{D}}}}}}}}}}{4\pi }\int\nolimits_{-\infty }^{\infty }\left(\frac{3{x}^{2}}{{r}^{5}}-\frac{1}{{r}^{3}}\right)dy=\frac{{\mu }_{0}{M}^{1{{{{{{{\rm{D}}}}}}}}}}{2\pi }\frac{({x}^{2}-{d}^{2})}{{({x}^{2}+{d}^{2})}^{2}}\\ {B}_{z}^{1{{{{{{{\rm{D}}}}}}}}}= \,\frac{{\mu }_{0}{M}^{1{{{{{{{\rm{D}}}}}}}}}}{4\pi }\int\nolimits_{-\infty }^{\infty }\frac{3xd}{{r}^{5}}dy=\frac{{\mu }_{0}{M}^{1{{{{{{{\rm{D}}}}}}}}}}{2\pi }\frac{2xd}{{({x}^{2}+{d}^{2})}^{2}}$$

where *r*^2^ = *x*^2^ + *y*^2^ + *d*^2^.

### Susceptibility fitting for Pd and Bi discs

Susceptibility fits followed the assumption that the stray field produced by a para- or diamagnetic sample is identical to that of a homogeneously magnetized body with a magnetization magnitude of *M* = *χ*_Pd/Bi_*B*_ext_/μ_0_ and a magnetization vector that is parallel to the external polarizing field. The stray field produced by this magnetization is **B**(**r**) = − μ_0_ ∇ *ϕ*_mag_(**r**) where *ϕ*_mag_(**r**) is the magnetic potential. We computed the stray field using the *k*-space method of ref. ^71^ and fitted the gradient image to the numerically computed gradient fields. In the fitting procedure we fixed *x*_osc_ = 69 nm, *α* = 180^∘^ (determined by the tip calibration) and sample thickness *t* = 50 nm while the standoff distance *d*, magnetization magnitude *M*, angles *θ* and *φ*, circle radius and position are free parameters in the fit. The susceptibilities are computed as *χ*_Pd/Bi_ = μ_0_*M*/*B*_ext_ and the fitted magnetizations were *M*_Pd_ = 18.6 ± 0.6 A/m and *M*_Bi_ = −4.4 ± 0.4 A/m.

## Supplementary information

Supplementary Information

## Data Availability

The data that support the findings of this study are available from the corresponding author upon request.
