# Supplementary material for: Scanning gradiometry with a single spin quantum magnetometer
Source: Nat Commun. 2022 Jun 29;13:3761. doi: 10.1038/s41467-022-31454-6 (PMC9243102; doi:10.1038/s41467-022-31454-6)
Supplement: Supplementary file 1 — Supplementary Information [file 41467_2022_31454_MOESM1_ESM.pdf]

Supplementary Information:
Scanning gradiometry with a single spin quantum magnetometer

W. S. Huxter[1,†], M. L. Palm[1,†], M. L. Davis[1,†], P. Welter[1],
C.-H. Lambert[2], M. Trassin[3], and C. L. Degen[1,4]

[1] *Department of Physics, ETH Zurich,*
Otto Stern Weg 1, 8093 Zurich, Switzerland.

[2] *Department of Materials, ETH Zurich,*
Hönggerbergring 64, 8093 Zurich, Switzerland.

[3] *Department of Materials, ETH Zurich,*
Vladimir Prelog Weg 1-5/10, 8093 Zurich, Switzerland. and

[4] *Quantum Center, ETH Zurich, 8093 Zurich, Switzerland.*[*]

(Dated: June 14, 2022)

SUPPLEMENTARY NOTE 1: PHASE ACCUMULATION FOR DIFFERENT SENSING SCHEMES

1.1. Generating an ac signal from a static field

When the tuning fork (TF) is stationary (no ac driving voltage applied) there are no ac magnetic fields to be observed by the NV center. However, as the NV moves in space it experiences a changing magnetic field that can be approximated with a Taylor expansion. For simplicity we can assume that the NV moves along the x-direction, although in general it oscillates along some angle α in the 2D plane. Up to second order, the magnetic field around x_0 can be approximated by:

$$B(x) \approx B(x_0) + \left.\frac{\partial B}{\partial x}\right|_{x=x_0} (x - x_0) + \frac{1}{2} \left.\frac{\partial^2 B}{\partial x^2}\right|_{x=x_0} (x - x_0)^2 \ . \tag{1}$$

When an ac driving voltage is applied to the TF the NV oscillates sinusoidally, in general by $x(t) = x_0 + x_{\text{osc}} \sin(2\pi f_{\text{TF}} t + \delta)$, and thus experiences an ac magnetic field:

$$B(t) \approx B_0 + B_1 \sin(2\pi f_{\text{TF}} t + \delta) + B_2 \sin^2(2\pi f_{\text{TF}} t + \delta) \tag{2}$$

Here, $B_1 = x_{\text{osc}} \left.\frac{\partial B}{\partial x}\right|_{x=x_0}$, $B_2 = \frac{x_{\text{osc}}^2}{2} \left.\frac{\partial^2 B}{\partial x^2}\right|_{x=x_0}$, f_{TF} is the driving frequency and δ represents a phase mismatch between the driving signal and the TF motion. In order to optimally sense the ac fields (with $\phi = \int_0^\tau \gamma_{\text{e}} g(t) B(t) dt$) the pulse sequences and TF motion must be synchronized.

1.2. Ramsey pulse sequence

During a Ramsey pulse sequence the TF drive is off, the modulation function $g(t)$ is constant ($g(t) = 1$), and $B(t) = B_0$ so the accumulated phase is:

$$\phi = \int_0^\tau \gamma_{\text{e}} g(t) B(t) dt = \gamma_{\text{e}} B_0 \tau \tag{3}$$

1.3. CPMG-1 gradient pulse sequence

Centering the pulse sequence around a time t_0 gives:

$$\phi = \int_{t_0-\tau/2}^{t_0+\tau/2} \gamma_{\text{e}} g(t) B(t) dt \tag{4}$$

* degenc@ethz.ch; † These authors contributed equally.

where $g(t) = +1$ for $t < t_0$ and $g(t) = -1$ for $t > t_0$. Computing the integral given by Eq. 4 gives:

$$\phi_1 = -\gamma_e B_1 \frac{2\cos(2\pi f_{\mathrm{TF}} t_0 + \delta)\sin^2(\pi f_{\mathrm{TF}}\tau/2)}{\pi f_{\mathrm{TF}}} - \gamma_e B_2 \frac{\sin(4\pi f_{\mathrm{TF}} t_0 + 2\delta)\sin^2(\pi f_{\mathrm{TF}}\tau)}{2\pi f_{\mathrm{TF}}} \tag{5}$$

In general, ϕ_1 depends on both the B_1 and B_2 fields, however, in practice we want to center the pulse sequence directly at the mid point of the TF oscillation. Setting t_0 such that $\sin(2\pi f_{\mathrm{TF}} t_0 + \delta) = 0$ implies that $2\pi f_{\mathrm{TF}} t_0 + \delta = n\pi$ (with $n \in \mathbb{Z}$). As a result $\cos(2\pi f_{\mathrm{TF}} t_0 + \delta) = (-1)^n$ and ϕ_1 depends solely on B_1:

$$\phi_1 = (-1)^{n+1}\gamma_e B_1 \frac{2\sin^2(\pi f_{\mathrm{TF}}\tau/2)}{\pi f_{\mathrm{TF}}} \tag{6}$$

Another interpretation of the $(-1)^n$ term is that it results from centering on the positive or negative zero-crossing of the TF motion around the position x_0.

1.4. CPMG-2 gradient pulse sequence

Following the derivation for the CPMG-1 pulse sequence, the modulation function for the CPMG-2 pulse sequence takes the form:

$$g(t) = \begin{cases} 1 & \text{if } t_0 - \tau/2 < t < t_0 - \tau/4 \\ -1 & \text{if } t_0 - \tau/4 < t < t_0 + \tau/4 \\ 1 & \text{if } t_0 + \tau/4 < t < t_0 + \tau/2 \end{cases} \tag{7}$$

The accumulated CPMG-2 phase is:

$$\begin{aligned} \phi_2 = -\gamma_e B_1 &\frac{4\sin(2\pi f_{\mathrm{TF}} t_0 + \delta)\sin^2(\pi f_{\mathrm{TF}}\tau/4)\sin(\pi f_{\mathrm{TF}}\tau/2)}{\pi f_{\mathrm{TF}}} \\ + \gamma_e B_2 &\frac{\cos(4\pi f_{\mathrm{TF}} t_0 + 2\delta)\sin^2(\pi f_{\mathrm{TF}}\tau/2)\sin(\pi f_{\mathrm{TF}}\tau)}{\pi f_{\mathrm{TF}}} \end{aligned} \tag{8}$$

Again, the accumulated phase depends on both B_1 and B_2. Centering the pulse sequence with the same t_0 value as above produces a phase that only depends on B_2:

$$\phi_2 = \gamma_e B_2 \frac{\sin^2(\pi f_{\mathrm{TF}}\tau/2)\sin(\pi f_{\mathrm{TF}}\tau)}{\pi f_{\mathrm{TF}}} \tag{9}$$

1.5. Multi-period sensing

When sensing across an integer number of periods, τ takes on integer multiples of half the period ($\tau = \frac{n}{2f}$ for $n \in \mathbb{N}$, since there are two π-pulses per period). It is simplest to derive the phase by first re-casting the magnetic field with cosine terms. Letting ($\delta' = \delta - \pi/2$):

$$B(t) \approx B_0 + B_1 \cos(2\pi f_{\mathrm{TF}} t + \delta') + B_2 \cos^2(2\pi f_{\mathrm{TF}} t + \delta') \tag{10}$$

A general modulation function that applies two π-pulses per oscillation period takes the following form:

$$g(t) = (-1)^{\lfloor 2 f_{\mathrm{TF}} t + 1/2 \rfloor} \tag{11}$$

The $+1/2$ term offsets the ± 1 switching so that $g(t)$ is in phase with a cosine signal. From this function we can define two modulation functions that can selectively sense for B_1 and B_2 by correcting for the phase mismatch:

$$g_1(t) = g\left(t + \frac{\delta'}{2\pi f_{\mathrm{TF}}}\right) \tag{12}$$

$$g_2(t) = g\left(2\left[t + \frac{\delta'}{2\pi f_{\mathrm{TF}}}\right]\right) \tag{13}$$

$g_1(t)$ demodulates $B(t)$ at $1 f_{\mathrm{TF}}$ and $g_2(t)$ demodulates $B(t)$ at $2 f_{\mathrm{TF}}$. The quantum phase accumulated for the $g_1(t)$ and $g_2(t)$ modulation functions are:

$$\phi_1 = \int_0^\tau \gamma_{\mathrm{e}} g_1(t) B(t) dt = 2\gamma_{\mathrm{e}} B_1 \tau / \pi \tag{14}$$

$$\phi_2 = \int_0^\tau \gamma_{\mathrm{e}} g_2(t) B(t) dt = \gamma_{\mathrm{e}} B_2 \tau / \pi \tag{15}$$

SUPPLEMENTARY NOTE 2: THEORETICAL AND EXPERIMENTAL SENSITIVITY

2.1. Shot noise limited sensitivities

In a four-phase measurement the collected NV PL counts C_i are:

$$\begin{aligned} C_x &= C_0\left(1-\frac{\epsilon}{2}\left[1+e^{-\xi(\tau)}\cos\omega\tau\right]\right)\\ C_y &= C_0\left(1-\frac{\epsilon}{2}\left[1-e^{-\xi(\tau)}\sin\omega\tau\right]\right)\\ C_{-x} &= C_0\left(1-\frac{\epsilon}{2}\left[1-e^{-\xi(\tau)}\cos\omega\tau\right]\right)\\ C_{-y} &= C_0\left(1-\frac{\epsilon}{2}\left[1+e^{-\xi(\tau)}\sin\omega\tau\right]\right) \end{aligned} \tag{16}$$

where C_0 is the $m_s = 0$ state count number, ϵ is the Rabi contrast, $\xi(\tau)$ is a dephasing function dependent on the coherent lifetime of the NV and ω is the detuning frequency (relative to the resonance frequency). The photon count of the $m_s = \pm 1$ states is $C_0(1-\epsilon)$ and the quantum phase accumulated during the measurement is $\phi = \omega\tau$ and can be computed using the two argument arctangent function as:

$$\phi = \arctan\left(\frac{\pm(C_{-y}-C_y)}{\pm(C_{-x}-C_x)}\right), \tag{17}$$

where the $\pm$ in the numerator (denominator) is taken for even or odd number of π_y-pulses (π_x-pulses). At the point of best sensitivity, a small change in quantum phase $\delta\phi$ results in a small change in the collected counts δC:

$$\delta C = \frac{C_0\epsilon e^{-\xi(\tau)}}{2}\delta\phi \tag{18}$$

The total counts $C_0 = I_0 t_{\mathrm{acq}} T/t_{\mathrm{s}}$ comes from the count rate I_0, the photon acquisition time of the avalanche photo diode t_{acq}, the total measurement time (or tip dwell time) T, and the sampling time for a single measurement t_{s}. The time for a single measurement is longer than the phase accumulation time due to laser initialization/readout and other overhead times ($t_{\mathrm{s}} = \tau + t_{\mathrm{d}}$ where t_{d} is the "dead" time). The shot noise limited signal-to-noise ratio can be calculated as $\mathrm{SNR} = \delta C/\sqrt{C_{\mathrm{avg}}}$, where $C_{\mathrm{avg}} = (C_x + C_y + C_{-x} + C_{-y})/4 = C_0\,(1-\epsilon/2)$. The minimal detectable quantum phase is detected at unity SNR and is:

$$\delta\phi_{\mathrm{min}} = \frac{2}{\epsilon e^{-\xi(\tau)}}\sqrt{\frac{(1-\frac{\epsilon}{2})t_{\mathrm{s}}}{I_0 t_{\mathrm{acq}} T}} \tag{19}$$

The minimal detectable magnetic field $\delta B_{\min}$ depends on the functional relationship between ϕ and the magnetic field. Additionally, a factor of $\sqrt{2}$ is needed to correct for the time spent measuring non-optimally. For example, when the signals C_y and C_{-y} are measured most sensitively, the signals C_x and C_{-x} are insensitive. This loss of sensitivity, however, comes with a trade-off as the four-phase measurement scheme provides a constant sensitivity through the entire $[-\pi;\pi)$ range of values.

2.2. Ramsey sensitivity

Using Eq. 3 and a dephasing function of $\xi(\tau) = (\tau/T_2^*)^a$ where a is between 1 and 3 gives a minimal detectable B-field of:

$$\delta B_{0,\min} = \frac{2}{\gamma_e \tau \epsilon e^{-\left(\tau/T_2^*\right)^a}} \sqrt{\frac{(1-\frac{\epsilon}{2})t_{\mathrm{s}}}{I_0 t_{\mathrm{acq}} T}} \tag{20}$$

The four-phase sensitivity can be calculated by setting the total measurement time to one second and including the factor of $\sqrt{2}$.

$$\eta_{B_0} = \frac{2}{\gamma_e \tau \epsilon e^{-\left(\tau/T_2^*\right)^a}} \sqrt{\frac{(2-\epsilon)t_{\mathrm{s}}}{I_0 t_{\mathrm{acq}}}} \tag{21}$$

2.3. CPMG-1 gradient sensitivity

For the CPMG-1 pulse sequence, ϕ_1 and B_1 field are related by Eq. 6. Effectively, the phase accumulation time τ in Eq. 21 is replaced by $2\sin^2(\pi f_{\mathrm{TF}}\tau/2)/(\pi f_{\mathrm{TF}})$ and t_{s} is replaced by $\lceil t_{\mathrm{s}} f_{\mathrm{TF}} \rceil / f_{\mathrm{TF}}$. The ceiling term is included since sampling times longer than the period of oscillation will force the measurement time to increase by an integer number of the period. Additionally, T_2^* is replaced by T_2, significantly prolonging the decay of the contrast. All together this gives a four-phase sensitivity of:

$$\eta_{B_1} = \frac{\pi}{\gamma_e \sin^2(\pi f_{\mathrm{TF}}\tau/2)\epsilon e^{-(\tau/T_2)^a}} \sqrt{\frac{(2-\epsilon)\lceil t_{\mathrm{s}} f_{\mathrm{TF}} \rceil f_{\mathrm{TF}}}{I_0 t_{\mathrm{acq}}}} \tag{22}$$

2.4. CPMG-2 gradient sensitivity

Following Eq. 9 and 21, the four-phase sensitivity is:

$$\eta_{B_2} = \frac{2\pi}{\gamma_e \sin^2(\pi f_{\mathrm{TF}}\tau/2)\sin(\pi f_{\mathrm{TF}}\tau)\epsilon e^{-\left(\tau/T_2^{CPMG-2}\right)^a}} \sqrt{\frac{(2-\epsilon)\lceil t_{\mathrm{s}} f_{\mathrm{TF}} \rceil f_{\mathrm{TF}}}{I_0 t_{\mathrm{acq}}}} \tag{23}$$

2.5. Multi-period gradient sensitivity

Measuring across multiple oscillation periods will results in $\tau = \frac{n}{2f_{\mathrm{TF}}}$ for sensing ϕ_1 or $\tau = \frac{n}{4f_{\mathrm{TF}}}$ for sensing ϕ_2. When setting the number of pulses to match an integer number of periods the time for a single measurement becomes $\lceil t_{\mathrm{s}} f_{\mathrm{TF}} \rceil / f_{\mathrm{TF}} = \tau + \lceil t_{\mathrm{d}} f_{\mathrm{TF}} \rceil / f_{\mathrm{TF}}$ and the dephasing function takes the form $\xi(\tau, n) = -\left(\frac{\tau}{T_2(n)}\right)^a = -\left(\frac{\tau}{T_2 n^{2/3}}\right)^3$ [1]. The resulting four-phase sensitivities become:

$$\eta_{B_1} = \frac{\pi}{\gamma_e \tau \epsilon e^{-\left(\tau/T_2 n^{2/3}\right)^3}} \sqrt{\frac{(2-\epsilon)(\tau + \lceil t_{\mathrm{d}} f_{\mathrm{TF}} \rceil / f_{\mathrm{TF}})}{I_0 t_{\mathrm{acq}}}} \tag{24}$$

$$\eta_{B_2} = \frac{2\pi}{\gamma_e \tau \epsilon e^{-\left(\tau/T_2 n^{2/3}\right)^3}} \sqrt{\frac{(2-\epsilon)(\tau + \lceil t_{\mathrm{d}} f_{\mathrm{TF}} \rceil / f_{\mathrm{TF}})}{I_0 t_{\mathrm{acq}}}} \tag{25}$$

2.6. Comparison of theoretical and experimental sensitivities

Supplementary Fig. 1a-b compares theoretical estimates with experimentally estimated sensitivities. For the theoretical curves we used the NV probe parameters of $\epsilon = 0.26$, $I_0 = 550\,\mathrm{kcnts/s}$, $T_2^* = 2.7\ \mu\mathrm{s}$, $T_2 = 31\ \mu\mathrm{s}$ and $T_2(n) = 31 n^{2/3}\ \mu\mathrm{s}$. Pulse sequence parameters of $t_{\mathrm{acq}} = 300\,\mathrm{ns}$ and $t_d = 3\ \mu\mathrm{s}$ were used and $a = 1$ for single-period sensing schemes. The tuning fork frequencies were 32 kHz, 196 kHz, and 521 kHz for the first, second and third TF resonances (see Supplementary Fig. 3 in Supplementary Note 4). Supplementary Fig. 1a displays the calculated sensitivity for the Ramsey, gradient CPMG-1, gradient CPMG-2 and traditional ac CPMG-1 sensitivities for a sinusodial signal [2]. Experimentally estimated sensitivities, extracted from images shown in the main text and supplemental, are indicated with stars. While the gradiometry sensitivities match expectations well, the experimental Ramsey sensitivity is significantly hindered by slow field drifts. Overall, a gain in sensitivity by an order of magnitude is experimentally achieved. Additionally, when optimized, the gradient CPMG-1 pulse sequence is as sensitive as the normal ac sensing protocol, demonstrating that scanning gradiometry provides ac sensitivities for dc samples. In Supplementary Fig. 1b theoretical sensitivities are shown for the multi-period gradient scheme. Individual points are plotted, in steps of four π-pulses. By comparison to Supplementary Fig. 1a the multi-period technique can improve the sensitivity much further by exploiting dynamical decoupling and sensitivities approaching $10\,\mathrm{nT}/\sqrt{\mathrm{Hz}}$ are feasible. The experimentally determined sensitivities (stars) do not match the theoretical expectations mainly

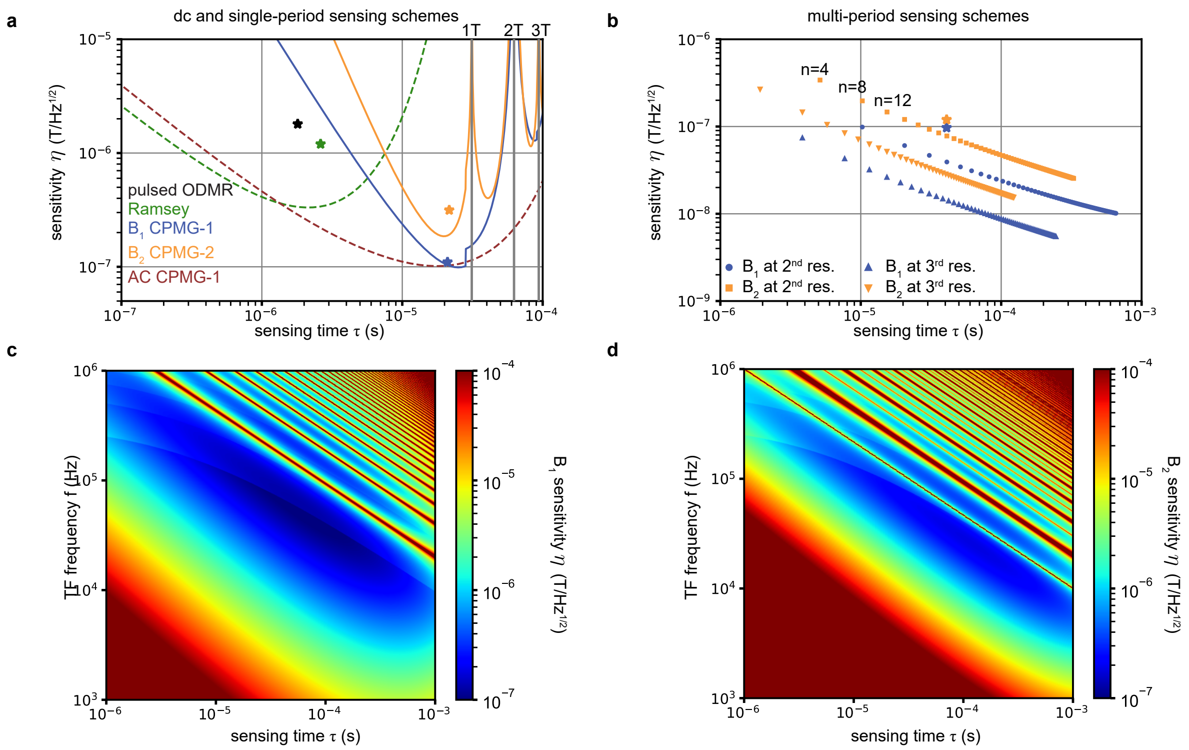

Supplementary Figure 1. **Theoretical and experimental sensitivities.** **a**, Sensitivities for dc and ac sensing sequences and single period gradiometry schemes. The pulsed ODMR [3] data point is estimated on Fig. 4b of the main text and the π-pulse duration is taken as the sensing time. The Ramsey data point is estimated on Supplementary Fig. 2b. The CPMG-1 and CPMG-2 data points are estimated from Fig. 3c of the main text. Vertical gray lines denote multiples of the periods ($1T$, $2T$, etc.). **b**, Sensitivities for multi-period scanning gradiometry schemes. The second and third resonance were set to 196 kHz and 521 kHz. Here, every point represents multiple of four π-pulses. The experimental data points are estimated from Supplementary Fig. 2d. Experimentally determined sensitivities are indicated as stars. **c-d**, Two-dimensional sensitivity plots of the single period gradiometry scheme as a function of tuning fork frequency f_{TF} and sensing time τ. In panels **a**, **c** and **d** discontinuities in the sensitivity are visible as a result of the ceiling term ($\lceil t_{\mathrm{s}} f_{\mathrm{TF}} \rceil$) jumping from n to $n+1$ sensing periods. Details surrounding the parameters values, such as contrast, are found in the text.

because this particular NV center was limited by T_1 and $T_2(n)$ did not scale with $n^{2/3}$ [1]. In Supplementary Fig. 1c-d it is possible to find B_1 sensitivities nearing $100\,\mathrm{nT}/\sqrt{\mathrm{Hz}}$ and B_2 sensitivities nearing $300\,\mathrm{nT}/\sqrt{\mathrm{Hz}}$ across a wide span of frequencies.

SUPPLEMENTARY NOTE 3: ADDITIONAL MEASUREMENTS OF CURRENTS IN GRAPHENE

Following the magnetometry images displayed in Fig. 3c of the main text, Supplementary Fig. 2a-b shows an alternative B_0 image acquired with a four-phase Ramsey technique. This image shows similar character as the pulsed ODMR image in the main text – low signal-to-noise and large magnetic drifts. Additionally in Supplementary Fig. 2c-d, gradiometry images are shown that demonstrate multi-period imaging at the second resonance mode of the TF. We show a B_1 image that was acquired using 16 π-pulses (XY16 pulse sequence) and a B_2 image that was acquired using 32 π-pulses (XY16-2 pulse sequence). In both cases the signal was acquired over 8 periods of oscillation, making the sensing time $8/f_{\mathrm{TF}} \approx 41$ μs.

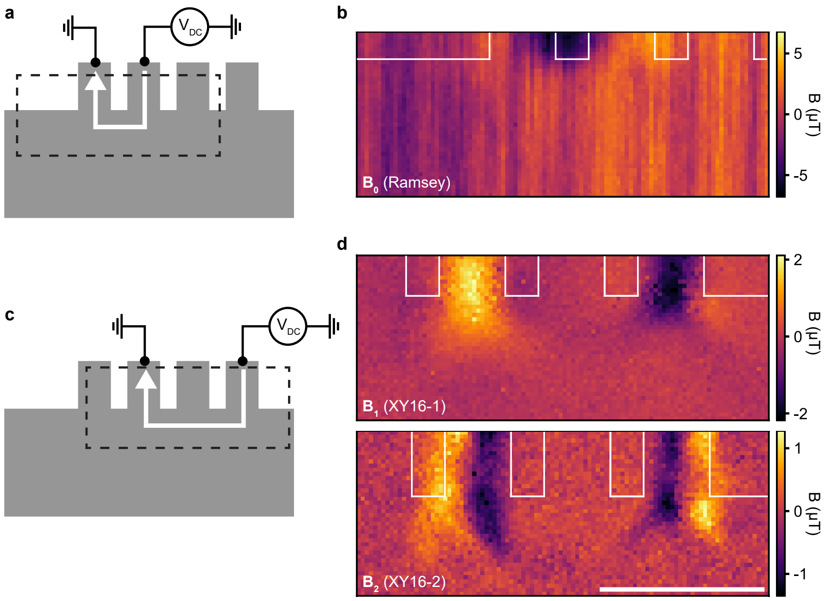

Supplementary Figure 2. **Ramsey and Multi-period scanning gradiometry.** **a**, Device geometry with contacts and current flow ($I_{\mathrm{dc}} \approx 5.3$ μA) identical to Fig. 3b of the main text. **b** Alternative B_0 measurement made with a four-phase Ramsey imaging scheme. 30 s dwell time per pixel. **c**, Device geometry with different contacts, indicating current flow for the images in panel **d**. A dc current of $I_{\mathrm{dc}} \approx 4.1$ μA was applied between the contacts. **d**, B_1 and B_2 images measured at the second mechanical resonance (~ 196 kHz, see Supplementary Fig. 3) with XY16-1 and XY16-2 pulse sequences. 15 s (top) and 17.5 s (bottom) dwell time per pixel. Scale bar is 1 μm.

SUPPLEMENTARY NOTE 4: CALIBRATION MEASUREMENTS

4.1. Trigger delay calibration

The CPMG-1 gradient phase depends on the trigger delay t_0 with a cosine dependence, assuming a negligible contribution from the B_2 term (see Eq. 5). This delay must be calibrated for every tip and TF frequency in order to cancel the phase mismatch δ (see Eq. 2). Examples of this calibration are shown in Supplementary Fig. 3a for the first three mechanical shear-mode resonances of a TF (recorded 20 nm retracted from the surface). In the calibration measurement the phase is measured as a function of trigger delay at a sample location where there is a gradient signal. This results in an oscillating phase that can be fitted with the amplitude and phase shift as free parameters ($\phi = \phi_0 \cos(2\pi f_{\mathrm{TF}} t_0 + \delta)$). The selected trigger delay used while imaging is the value which maximizes the phase in the calibration sweep.

The corresponding resonance curves for the first three shear-modes are shown in Supplementary Fig. 3b. A Butterworth-Van-Dyke circuit model was used to fit the data via the complex admittance:

$$Y(f) = \frac{1}{R + \frac{1}{i2\pi f C_1} + i2\pi f L} + i2\pi f C_2 \tag{26}$$

Here R, C_1, and L are a resistor, capacitor, and inductor connected in series with a parasitic capacitance C_2 connected in parallel. The quality factor is $Q = \sqrt{L/(C_1 R^2)}$.

4.2. Photoluminescence oscillations

The process of extracting the phase in a trigger delay calibration measurement naturally leads to quantifying how the PL of the NV center oscillates as a function of time and drive strength. In Supplementary Fig. 4a-b the PL counts are shown with and without the mean subtracted. The oscillation in the mean PL counts (black trace in Supplementary Fig. 4a) is caused by the NV moving in the stationary laser path. Supplementary Fig. 4c and s4d show how changing the oscillation strength of the tuning fork affects the PL and phase. As the oscillation drive strength increases the PL of the NV is modulated more heavily. The form of the modulation is modeled by, and fitted to, an oscillating point inside a Gaussian laser beam [4]. The mean counts C_{m} take the form:

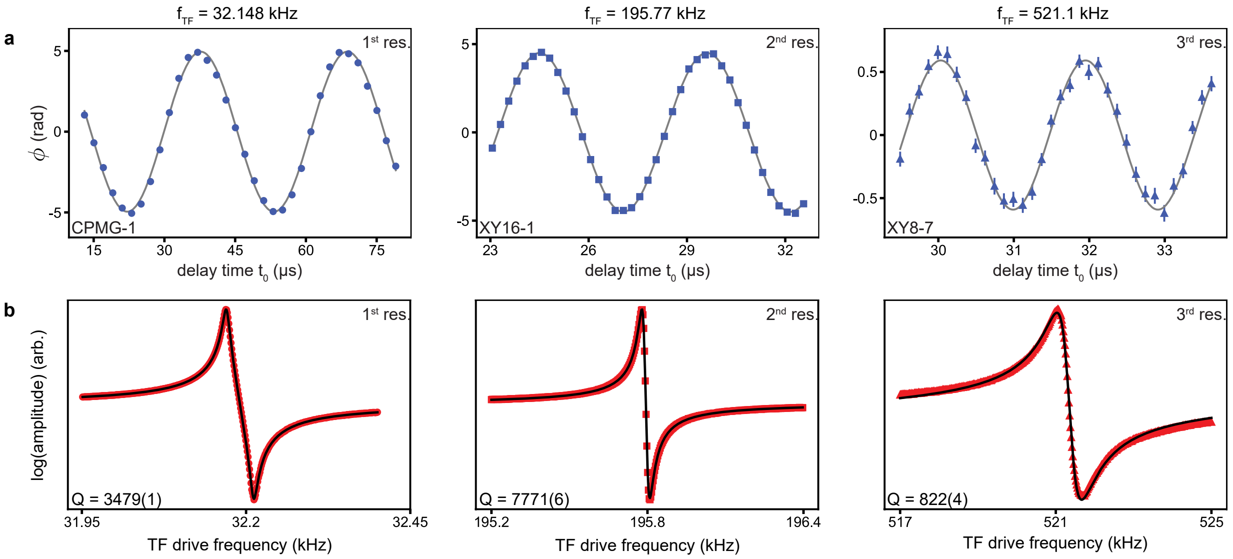

Supplementary Figure 3. **Trigger delay calibration curves and shear-mode resonances. a** Trigger delay calibration curves for the first three resonance modes of the tuning fork. The pulse sequence used is shown in the bottom left of the panel (1, 16, and 56 π-pulses are used for the first, second and third panels). Fits are described in the text and error bars are shot-noise propagated uncertainties. **b** The first three resonance modes of one of the tuning forks. Quality factors are displayed in the bottom left of the panel and the number in parenthesis is the uncertainty on the last digit. Fits are models by Eq. 26

$$C_{\rm m}(t_0) = C_{\rm m,0} \exp\left[-(A + B\sin(2\pi f_{\rm TF} t_0 + \beta))^2\right] \quad (27)$$

where $C_{\rm m,0}$ is the counts when stationary, A and B are shape parameters depending on the offset and strength of the oscillation, and β is a delay. As long as the PL does not drastically decrease while oscillating, the sensitivity of the measurement technique is largely unaffected (sensitivity scales with the square root of the counts). The phase computation is not affected by the PL oscillation, however the signal is proportional to the drive strength (see Supplementary Note 1).

4.3. Sensing time dependence on acquired phase

In Supplementary Fig. 5a-b line scans using the CPMG-1 and CPMG-2 pulse sequences show signal peaks on the order of 50 nm in width. The center of the shaded blue and

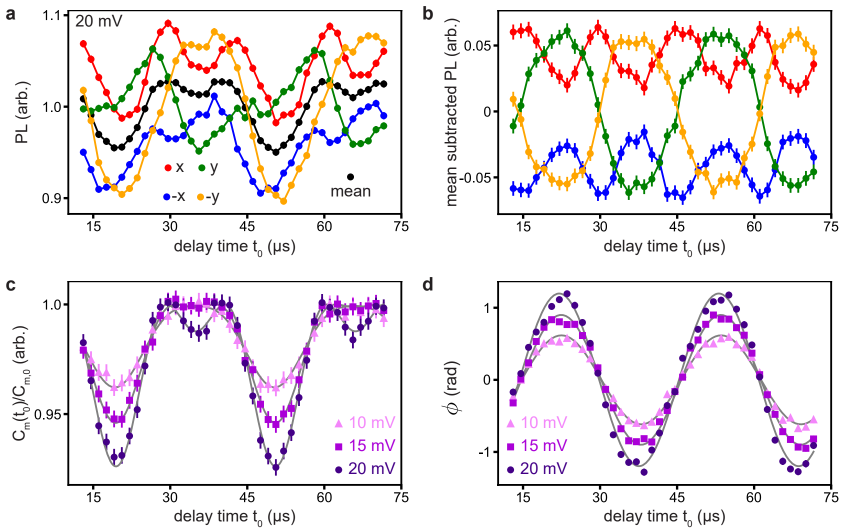

Supplementary Figure 4. **Characterization of photoluminescence oscillations.** **a**, Four-phase readouts, including the mean of the PL counts, for 20 mV tuning fork drive voltage. **b**, Same as in **a** but with mean subtraction. **c**, PL oscillations of the NV for different drive voltages. Fits are modeled by Eq. 27 **d**, Computed phase ϕ for different drive voltages. Fits are modeled the same as in Supplementary Fig. 3 and error bars are shot-noise propagated uncertainties.

orange regions indicate where the measurements in Supplementary Fig. 5c and Fig. 3a of the main text were measured. The widths of the shaded regions represent the peak-to-peak displacements ($2x_{\text{osc}}$) of the tip during the line scan. As shown in Fig. 3a, at larger driving amplitudes ($> 8\,\text{mV}$, corresponding to $> 74\,\text{nm}$ peak-to-peak oscillation) the signals begin to deviate from the linear and quadratic growth as a result of the Taylor approximation breakdown. In Supplementary Fig. 5c the CPMG-1 and CPMG-2 phases were measured as a function of sensing time τ and were fitted to $\phi_1 = \phi_0 \sin^2(\pi f_{\text{TF}}\tau/2)$ and $\phi_2 = \phi_0 \sin^2(\pi f_{\text{TF}}\tau/2)\sin(\pi f_{\text{TF}}\tau)$, respectively. Both data sets agree nicely with the predicted theory. The CPMG-1 phase continually increases with $\tau < T$ while the CPMG-2 sequence reaches a maximum phase at $\tau = 2/(3f_{\text{TF}})$. Increasing the sensing time beyond two thirds of the period causes the phase accumulated at different portions of the pulse sequence to cancel, resulting in less overall phase.

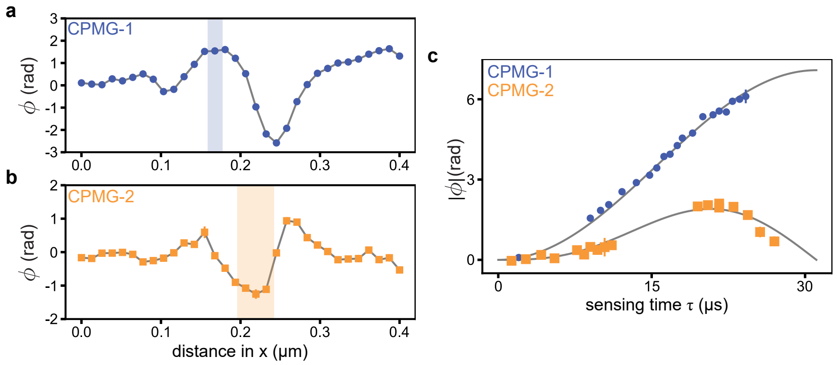

Supplementary Figure 5. **Sensing time characterization. a-b**, One dimensional line scans of CPMG-1 and CPMG-2 measurements recorded over the same region. **c**, Phase versus sensing time for CPMG-1 and CPMG-2 sensing sequences. Fits are discussed in the text and error bars are shot-noise propagated uncertainties..

4.4. Oscillation amplitude and angle using least-squares minimization

Here we estimate the real space oscillation amplitude x_{osc} and oscillation angle α by processing static field (B_0) and first derivative (B_1) images acquired over the same region with a least-squares minimization scheme. In this analysis the static field image B_0 is numerically differentiated and compared to the gradient image B_1 using the following cost function:

$$\zeta^2 = \frac{\sum[B_1 - x_{\text{osc}} D_\alpha(B_0)]^2}{\sigma_1^2 + x_{\text{osc}}^2 \bar{\sigma_0}^2} \tag{28}$$

In the above equation the sum is taken over all pixels and $D_\alpha = \cos\alpha \frac{d}{dx} + \sin\alpha \frac{d}{dy}$ is the discrete directional derivative. σ_1 and $\bar{\sigma_0}$ are the estimated standard deviations of B_1 (in units of tesla) and $D_\alpha(B_0)$ (in units of tesla per meter), respectively. Since we define $B_1 = x_{\text{osc}} \partial_\alpha B_0$, the correct oscillation angle and amplitude will minimize ζ. There are two additional parameters involved in the fit, shifts in x and y, since the B_0 and B_1 images are taken at different times and will generally be shifted relative to each other. This can be avoided by simultaneously measuring the B_0 and B_1 fields at each pixel. Differentiating ζ^2 with respect to x_{osc} and finding the zeros we get one solution which maximizes the cost and another which minimizes it. Letting $A = \sum \bar{\sigma_0}^2 D_\alpha(B_0) B_1$, $B = \sum(\sigma_1^2 D_\alpha(B_0)^2 - \bar{\sigma_0}^2 B_1^2)$, and $C = -\sum \sigma_1^2 D_\alpha(B_0) B_1$, we get the minimizing solution with $x_{\text{osc}} = (-B + \sqrt{B^2 - 4AC})/2A$.

We can also estimate the standard deviation on x_{osc} with standard error propagation:

$$\sigma_{x_{\text{osc}}}^2 = \sum \left[\left(\frac{\partial x_{\text{osc}}}{\partial (D_\alpha(B_0))} \right)^2 \bar{\sigma_0}^2 + \left(\frac{\partial x_{\text{osc}}}{\partial B_1} \right)^2 \sigma_1^2 \right] \tag{29}$$

where, for every pixel:

$$\frac{\partial x_{\text{osc}}}{\partial (D_\alpha(B_0))} = \frac{-\bar{\sigma_0}^2 B_1 x_{\text{osc}}^2 - 2\sigma_1^2 D_\alpha(B_0) x_{\text{osc}} + \sigma_1^2 B_1}{2Ax_{\text{osc}} + B} \tag{30}$$

$$\frac{\partial x_{\text{osc}}}{\partial B_1} = \frac{-\bar{\sigma_0}^2 D_\alpha(B_0) x_{\text{osc}}^2 + 2\bar{\sigma_0}^2 B_1 x_{\text{osc}} + \sigma_1^2 D_\alpha(B_0)}{2Ax_{\text{osc}} + B} \tag{31}$$

σ_1 can be estimated from experimental measurements alone, while $\bar{\sigma_0}$ can be estimated from the standard deviation of the B_0 image (σ_0, in units of tesla) and the numerical differentiation process which depends on the pixel resolutions Δ_x and Δ_y and oscillation angle α:

$$\bar{\sigma_0} = \frac{\sigma_0}{\sqrt{2}} \sqrt{\left(\frac{\cos\alpha}{\Delta_x} \right)^2 + \left(\frac{\sin\alpha}{\Delta_y} \right)^2} = \frac{\sigma_0}{\sqrt{2}\Delta} \tag{32}$$

The last equality is for square pixel resolutions ($\Delta_x = \Delta_y = \Delta$). Other changes to the estimated standard derivations must be considered from the linear pixel interpolation when trying to find the shifts in x and y.

An example of the fitting procedure is shown in Supplementary Fig. 6. From two images (Supplementary Fig. 6a-b), a series of fits for the x and y shifts are performed for different oscillation angles α. One example is shown in Supplementary Fig. 6d, where, for every pixel the cost function is already minimized with respect to x_{osc}. The minimum cost from this 2D grid is then plotted as a function of the oscillation angle α shown in Supplementary Fig. 6e. The angle α that minimizes this curve directly leads to an estimated amplitude x_{osc} and amplitude error shown in Supplementary Fig. 6f. The fit parameters from this process are $x_{\text{osc}} = 19.9 \pm 0.5\,\text{nm}$ and $\alpha = 3.14\,\text{rad}$ which corresponds to an oscillation along the x-axis. The processed B_0 image, $x_{\text{osc}} D_\alpha(B_0)$, using the optimized values is shown in Supplementary Fig. 6c. By comparing panels b and c of Supplementary Fig. 6 to each other, the higher quality of the B_1 image is evident as high-frequency noise (which is introduced by the differentiation process) obscures the signal in Supplementary Fig. 6c. This noise conceals most of the sharper image features and precludes any benefit from field drift suppression that differentiation might introduce.

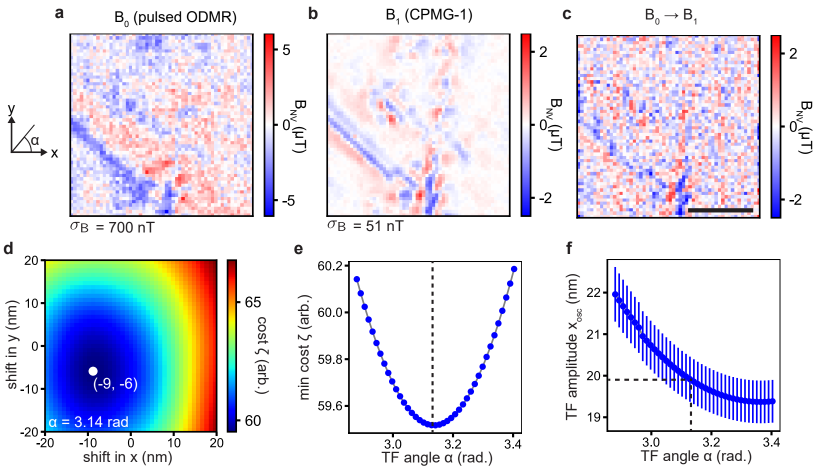

Supplementary Figure 6. **Determination of the oscillation angle and amplitude using B_0 and B_1 images. a-b**, Selected images for the least-squares fitting procedure. **c**, Transformation of the B_0 image using the optimized x_{osc} and α parameters. **d**, Cost function ζ for $\alpha = 3.14$ rad for different image translations in x and y. **e**, Minimum of the cost function ζ for different oscillation angles. The minimum of this curve is the selected oscillation angle. **f**, Optimized tuning fork amplitude for different oscillation angles. Error bars are described in the text. Scale bar is 500 mm.

4.5. Oscillation amplitude and angle using stroboscopic imaging

Here, we estimate the real space oscillation amplitude x_{osc} and oscillation angle α by measureing the relative shift of image features acquired along the oscillation of the tuning fork. Multiple B_0 images were collected simultaneously at different times along the oscillation of the tuning fork. In the different images the magnetic features are shifted with respect to each other. The stroboscopic data set is acquired by timing the pulsed sequences with the TF trigger signal, similar to scanning gradiometry. An example of this is shown in Supplementary Fig. 7a where six different pulsed ODMR images show the same central magnetic feature that shifts along the x-axis. From these shifts a physical path of the NV sensor can be fitted to a sinusoidal function. In the fit the frequency was set to the resonance frequency while the amplitude, offset and phase were free parameters. The results of these fits are shown in Supplementary Fig. 7b for three different sets of images with 5 mV, 15mV and 30 mV tuning fork drives. The amplitude extracted from these fits provides a direct relationship between the set TF drive voltage and the real space oscillation amplitude x_{osc}, shown

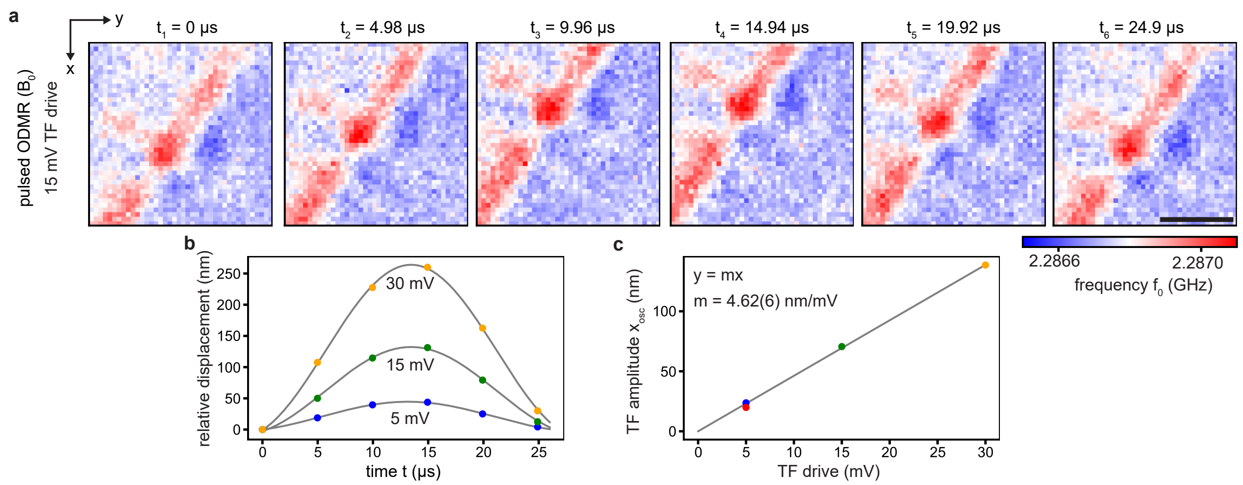

Supplementary Figure 7. **Determination of the TF drive-amplitude relationship using stroboscopic measurements.** **a**, A series of stroboscopic pulsed ODMR images taken over the polished Cr_2O_3 sample. Six pulsed ODMR measurements were taken throughout a single oscillation period, enabling the possibility to correlate the displacement of spatial magnetic features to the oscillation amplitude of the tuning fork. Scale bar is 200 nm. **b**, Sinusoidal fits between stroboscopic images for each set of images acquired with different TF drives. **c**, Amplitudes from panel **b** provide a direct relationship between the TF drive voltage and the oscillation amplitude, which is fitted to a linear function of the form $y = mx$. The red data point corresponds to the fit from the least-squares minimization scheme.

in Supplementary Fig. 7c. Within the region of measurement the relation appears to be linear. Note, no measurable shifts were measured in y, which indicates that the oscillation is along the x-axis. There is considerable agreement between both the least-squares and stroboscopic estimation methods.

SUPPLEMENTARY NOTE 5: GRADIENT MEASUREMENT WHILE IN CONTACT

Supplementary Fig. 8 displays trigger delay calibration curves at 50 nm, 20 nm, and 0 nm (in contact) above a sample. While the amplitude of the measured phase increases going from 50 nm to 20 nm nm as expected, the measurement taken in contact with the sample is much more complicated to understand. In contact two things occur: (i) x_{osc} decreases as part of the PID control feedback mechanism, reducing the gradient signal and (ii) an additional signal is picked up that appears to be a second derivative component. Interestingly, no second derivative signal was observed in panels a and b of Supplementary Fig. 8. For this reason we attribute the additional signal, which appears at twice the tuning fork frequency ($2f_{\text{TF}}$), to a time-dependent strain interaction experienced by the NV. Previous investigations have demonstrated that NV centers subject to sinusoidal time-dependent strain at frequency f can experience spin population fluctuations at both f and $2f$ [5]. Thus, to minimize strain interaction, scanning gradiometry images are measured with the tuning fork slightly retracted from the sample.

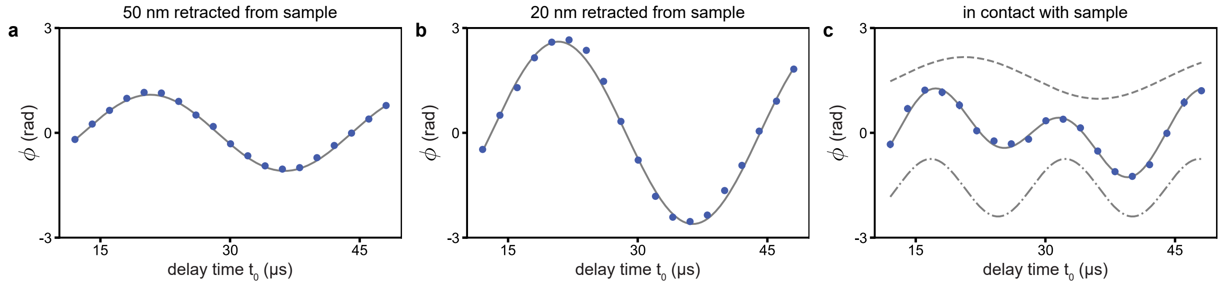

Supplementary Figure 8. **Delay curves as a function of height above the sample. a-b**, Delay curves taken 50 and 20 nm above the surface of the sample. Fitted functions are of the form $\phi_0 \cos(2\pi f t_0 + \delta)$. **c**, Delay curve taken in contact with the sample. The fitted function (solid gray) is composed of two oscillating functions (see Eq. 8) composed of $\phi_{0,1} \cos(2\pi f t_0 + \delta)$ (dashed gray) and $\phi_{0,2} \sin(4\pi f t_0 + 2\delta)$ (dot-dashed gray). The two components of the fit are shown shifted for clarity. Error bars are shot-noise propagated uncertainties.

SUPPLEMENTARY NOTE 6: VECTOR COMPONENT RECONSTRUCTION OF GRADIENT FIELDS

When applying a bias field along the anisotropy axis of the NV center, the NV measures the projection of the sample's magnetic field along that same anisotropy axis . Defining that axis as ($\mathbf{e} = [e_x, e_y, e_z] = [\sin\theta\cos\varphi, \sin\theta\sin\varphi, \cos\theta]$) we get $B_{\mathrm{NV}} = \mathbf{e}\cdot\mathbf{B} = e_x B_x + e_y B_y + e_z B_z$. Here, $[B_x, B_y, B_z]$ can take the form of the static or dynamic magnetic fields (B_0, B_1, or B_2). Thus, it is possible to reconstruct the vector components (in the lab frame) of the first and second spatial derivatives of the static magnetic field. This is possible since the curl of the magnetic field is zero (there are no magnetic sources at the location of the NV) which leads to $ik_x\hat{B}_y = ik_y\hat{B}_x$, $ik_x\hat{B}_z = \sqrt{k_x^2+k_y^2}\hat{B}_x$ and $ik_y\hat{B}_z = \sqrt{k_x^2+k_y^2}\hat{B}_y$ in k-space. The vector components are:

$$\begin{aligned}\hat{B}_x(k_x,k_y) &= \frac{ik_x\hat{B}_{NV}}{ie_xk_x + ie_yk_y + e_z\sqrt{k_x^2+k_y^2}}\\ \hat{B}_y(k_x,k_y) &= \frac{ik_y\hat{B}_{NV}}{ie_xk_x + ie_yk_y + e_z\sqrt{k_x^2+k_y^2}}\\ \hat{B}_z(k_x,k_y) &= \frac{\sqrt{k_x^2+k_y^2}\hat{B}_{NV}}{ie_xk_x + ie_yk_y + e_z\sqrt{k_x^2+k_y^2}}\end{aligned} \tag{33}$$

Taking the inverse Fourier transform produces the real space B_x, B_y, and B_z components. Examples of the vector component reconstruction of the first and second spatial derivatives are shown in Supplementary Fig. 9. Additionally, derivatives of the stray field produced by two infinitely long and infinitely thin current carrying wires along y are plotted in the B_x and B_z images for qualitative comparison and easier interpretation. Functionally, the static field components behave as $B_x(x) \propto \frac{-d}{(x-a)^2+d^2} + \frac{d}{(x+a)^2+d^2}$ and $B_z(x) \propto \frac{x-a}{(x-a)^2+d^2} - \frac{x+a}{(x+a)^2+d^2}$, where d is the distance in z above wires separated by a distance of $2a$.

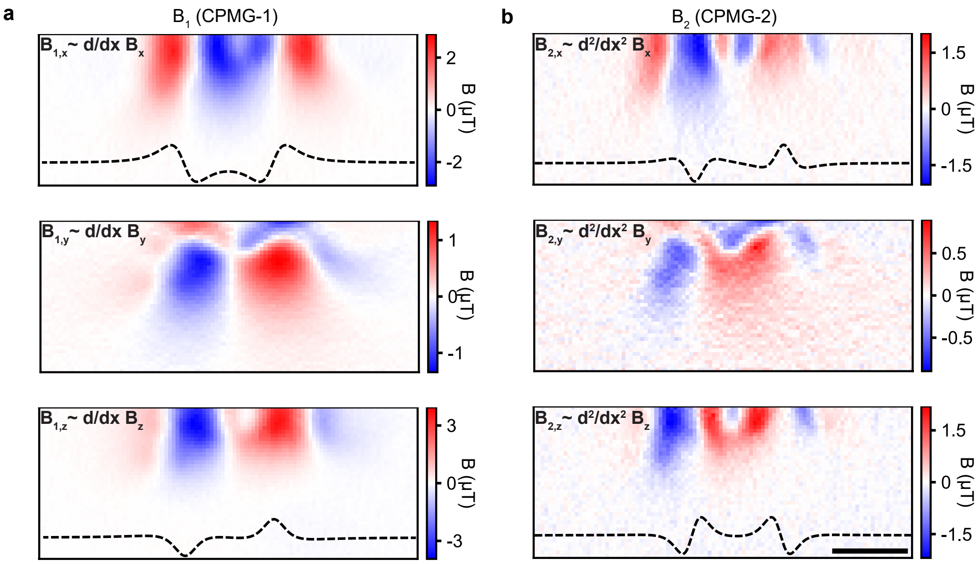

Supplementary Figure 9. **Vector reconstruction of the first and second spatial derivatives.** **a**, Lab frame vector components of the first spatial derivative, reconstructed with the data shown in Fig. 3c of the main text. **b**, same as in a, but with the second spatial derivative. Models with infinitely thin wires for the x and z components are added as a guide. For the reconstruction $(\theta, \varphi) = (50.5°, 267°)$ was used. Scale bar is 500 nm.

SUPPLEMENTARY NOTE 7: MAGNETIC FIELDS AND GRADIENTS FROM TOPOGRAPHY

Stray fields produced by topographic height changes can be modeled by the stray fields produced at the edges of two magnetic materials with different heights. For an antiferromagnet with a surface magnetization of σ_z pointing in the z-direction, a height change h in z at $x = 0$ produces the magnetic fields components:

$$\begin{aligned} B_x^{\text{step}} &= B_x(x,d) - B_x(x,d+h) = \frac{-\mu_0\sigma_z}{2\pi}\left(\frac{d}{x^2+d^2} - \frac{d+h}{x^2+(d+h)^2}\right) \\ B_y^{\text{step}} &= 0 \\ B_z^{\text{step}} &= B_z(x,d) - B_z(x,d+h) = \frac{\mu_0\sigma_z}{2\pi}\left(\frac{x}{x^2+d^2} - \frac{x}{x^2+(d+h)^2}\right) \end{aligned} \tag{34}$$

where the NV standoff distance is d, and the stray fields at a single edge are given by $B_x(x,z) = \frac{-\mu_0\sigma_z}{2\pi}\frac{z}{x^2+z^2}$, $B_y = 0$, and $B_z(x,z) = \frac{\mu_0\sigma_z}{2\pi}\frac{x}{x^2+z^2}$ [6]. In the limit of $h \ll d$ the expression can be simplified as:

$$\begin{aligned} B_x^{\text{step}} &= \frac{\mu_0\sigma_z h}{2\pi}\frac{(x^2-d^2)}{(x^2+d^2)^2} \\ B_y^{\text{step}} &= 0 \\ B_z^{\text{step}} &= \frac{\mu_0\sigma_z h}{2\pi}\frac{2xd}{(x^2+d^2)^2} \end{aligned} \tag{35}$$

Here, both the B_x and B_z components scale linearly with h. Supplementary Fig. 10a and 10b plot the absolute maximum of the magnetic field and gradient components as a function of the height change. For $h/d < 0.1$ the maxima are well described by the approximation of Eq.35. From Supplementary Fig. 10, static field signals of a few μT can be expected from topographic changes on the order of a few nanometers (Fig. 4b of the main text) and magnetic gradients $2-4$ T/m are expected from $0.2-0.5$ nm height changes (Fig. 4g and h of the main text). The agreement for small h in Supplementary Fig. 10 justifies the use of the approximation for fitting the mono- and diatomic step edge measurements in Fig. 4 of the main text. Additionally, it also demonstrates that the relative amplitudes of the measured signal (static or gradient field) are directly related to the relative height changes in the local topography.

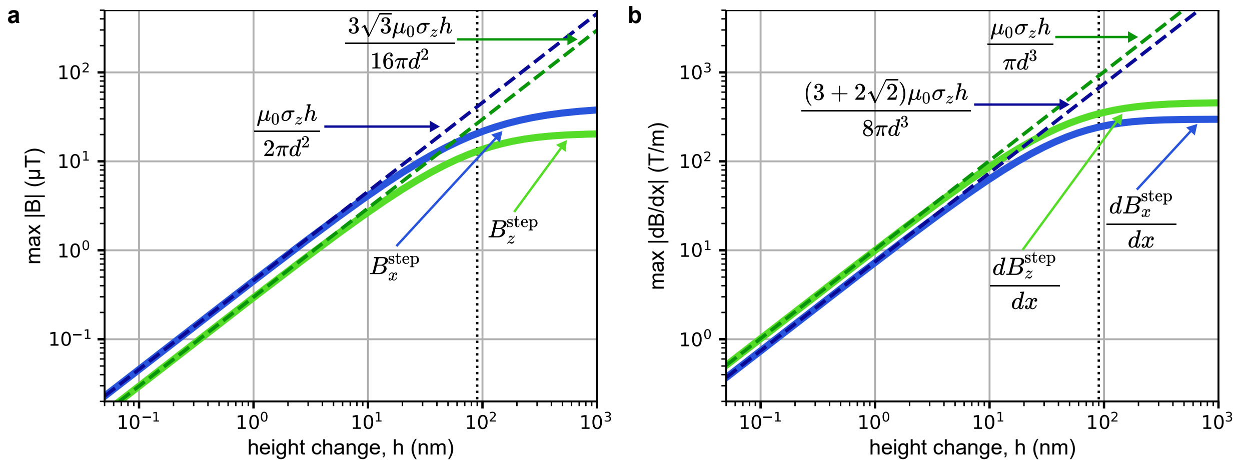

Supplementary Figure 10. **Calculated magnetic field and field gradients produced by topographic changes in height.** **a** Maxima of the B_x and B_z components of the magnetic field as a function of the height change (see text for details). Solid curves use Eq. 34 and dashed lines use Eq. 35. **b** Same calculation from **a**, but computed for the field gradients. In these plots, $\sigma_z = 2\,\mu_\mathrm{B}/\mathrm{nm}^2$ and $d = 90\,\mathrm{nm}$, closely following the fitted values from Fig. 4 of the main text. The standoff distance is indicated with a dotted black line.

SUPPLEMENTARY NOTE 8: ADDITIONAL MEASUREMENTS ON Cr_2O_3

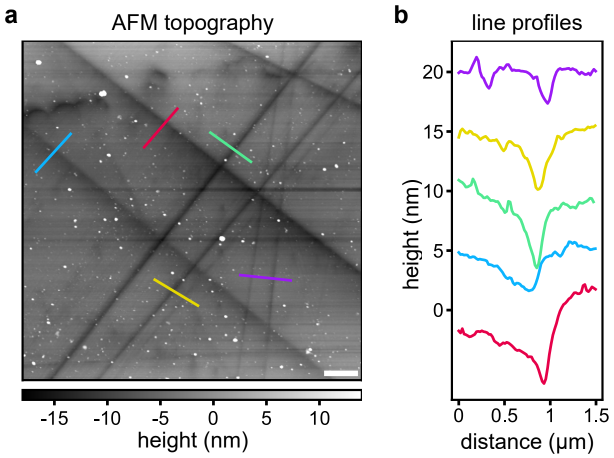

Supplementary Figure 11. **Atomic force microscopy on polished Cr_2O_3.** **a**, AFM topography image revealing polishing induced trenches. The surface roughness is ~ 3.5 nm-rms including the trenches and < 2 nm-rms excluding the trenches. **b**, Line profiles, extracted from **a** and separated by 5 nm, indicating that trenches are 2 to 5 nm in depth, and vary from 100 to 500 nm in width. Scale bar, 1 μm.

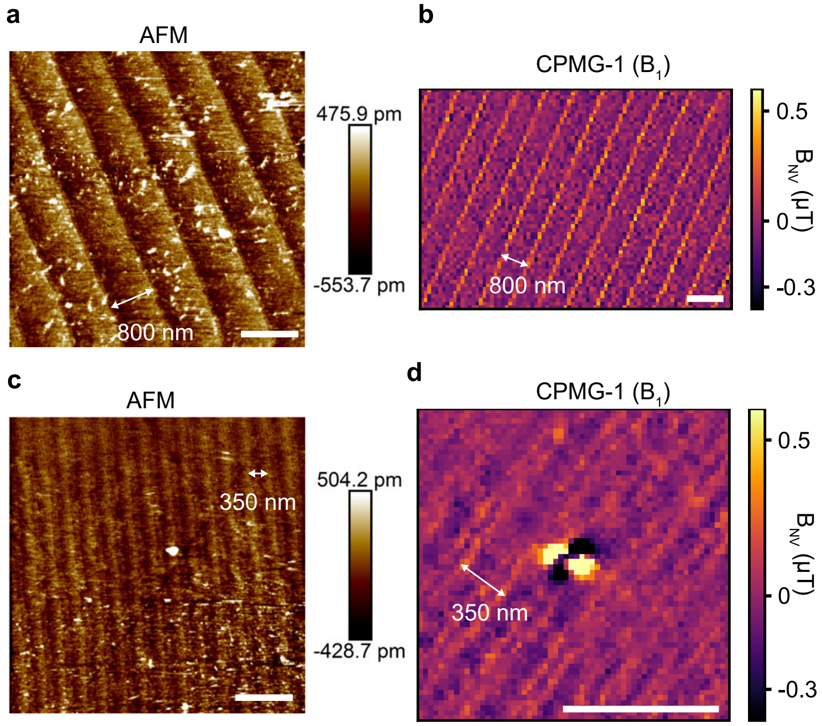

Supplementary Figure 12. **Atomic force microscopy and scanning gradiometry of Cr_2O_3 atomic steps.** **a**, AFM topography image revealing double atomic steps of the $Cr_2O_3(0001)$ surface with terrace $\sim 800\,\mathrm{nm}$ in size. **b**, B_1 image over double atomic steps with repeating features every $\sim 800\,\mathrm{nm}$. **c**, AFM topography image revealing single atomic steps of the $Cr_2O_3(0001)$ surface in a long-short terrace size pattern. Short terraces are $\sim 100\,\mathrm{nm}$ in size and long terraces are $\sim 250\,\mathrm{nm}$ in size. **d**, B_1 image over single atomic steps with the same short-long feature pattern. A magnetic defect is also visible. AFM images (measured separately after magnetic imaging) were recorded in the vicinity of the gradiometry images and feature different sample orientations. Dwell times per pixel in **b** and **d** are 5 s. Scale bars, 1 μm.

SUPPLEMENTARY NOTE 9: ADDITIONAL MEASUREMENTS ON Pd AND Bi MICRO-DISCS

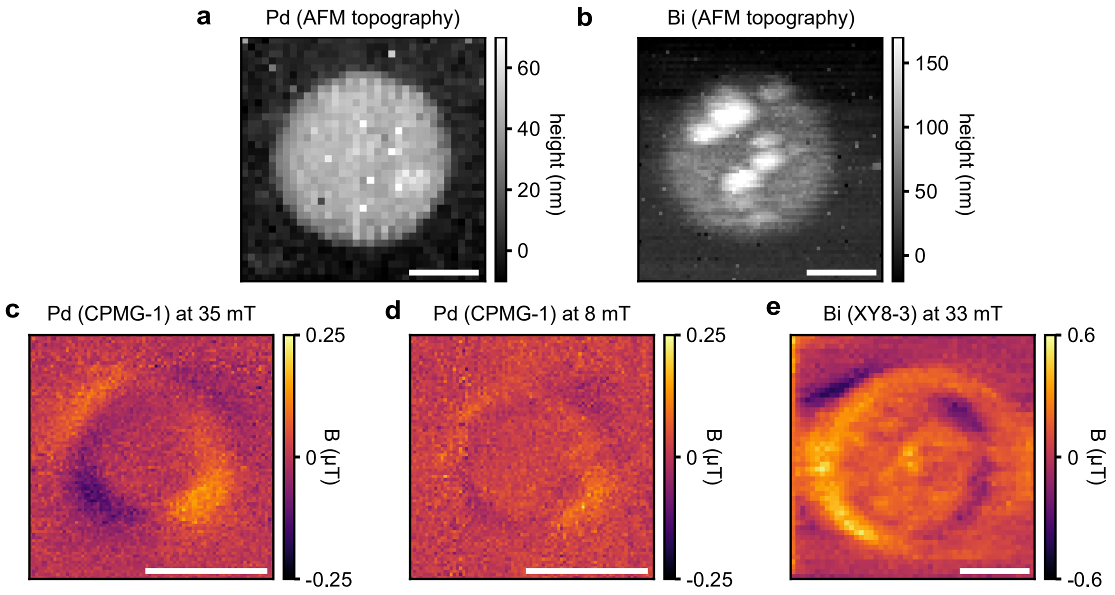

Supplementary Figure 13. **Additional images on para- and diamagnetic discs. a-b**, AFM topography, recorded with the scanning NV tip of the Pd and Bi samples shown in Fig. 5 of the main text. Topography variations are present on the Bi, but not on the Pd. **c-d**, Measured B_1 images of a 1-μm-diameter paramagnetic Pd disc at 35 and 8 mT external bias fields. The change in the signal strength is a result of the paramagnetism of Pd. **e**, Multi-period B_1 image of a 2-μm-diameter diamagnetic Bi disc at 33 mT with an XY8-3 pulse sequence (24 π-pulses over 12 oscillation periods). Offsets have been subtracted in panels **c** and **e**. Dwell times per pixel are 25 s in **c**, 30 s in **d**, and 16 s in **e**. Scale bars, 1 μm.

SUPPLEMENTARY NOTE 10: FIELD GRADIENTS AT DOMAIN WALLS

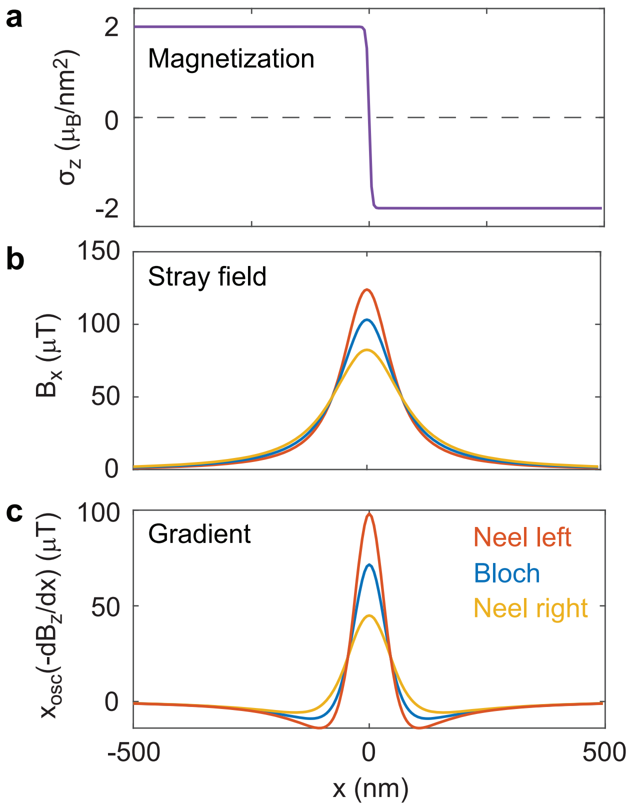

Supplementary Figure 14. **Calculated line scans from a 10-nm-wide domain wall in $\mathbf{Cr_2O_3}$** . **a** Profile of the surface magnetization component. **b** Computed B_x component of the magnetic stray field. **c** Computed $\partial B_z/\partial x$ component of the magnetic gradient. The magnetic gradient provides a narrower features size and better sensitivity towards the wall type (Bloch, Néel) compared to the stray field. The calculation placed the domain wall at $x = 0$, used an oscillation amplitude of $x_{\mathrm{osc}} = 50\,\mathrm{nm}$, stand-off distance of $d = 70\,\mathrm{nm}$, and followed the model in Ref. 7.

SUPPLEMENTARY REFERENCES

[1] G. de Lange, Z. Wang, D. Riste, V. Dobrovitski, and R. Hanson, Universal dynamical decoupling of a single solid-state spin from a spin bath, Science **330**, 60 (2010).

[2] M. Palm, W. Huxter, P. Welter, S. Ernst, P. Scheidegger, S. Diesch, K. Chang, P. Rickhaus, T. Taniguchi, K. Watanabe, K. Ensslin, and C. Degen, Imaging of submicroampere currents in bilayer graphene using a scanning diamond magnetometer, Physical Review Applied **17**, 10.1103/physrevapplied.17.054008 (2022).

[3] A. Dreau, M. Lesik, L. Rondin, P. Spinicelli, O. Arcizet, J. F. Roch, and V. Jacques, Avoiding power broadening in optically detected magnetic resonance of single NV defects for enhanced dc magnetic field sensitivity, Phys. Rev. B **84**, 195204 (2011).

[4] O. Arcizet, V. Jacques, A. Siria, P. Poncharal, P. Vincent, and S. Seidelin, A single nitrogen-vacancy defect coupled to a nanomechanical oscillator, Nature Physics **7**, 879 (2011).

[5] P. Ovartchaiyapong, K. W. Lee, B. A. Myers, and A. C. B. Jayich, Dynamic strain-mediated coupling of a single diamond spin to a mechanical resonator, Nature Communications **5**, 10.1038/ncomms5429 (2014).

[6] J. P. Tetienne, T. Hingant, L. J. Martinez, S. Rohart, A. Thiaville, L. H. Diez, K. Garcia, J. P. Adam, J. V. Kim, J. F. Roch, I. M. Miron, G. Gaudin, L. Vila, B. Ocker, D. Ravelosona, and V. Jacques, The nature of domain walls in ultrathin ferromagnets revealed by scanning nanomagnetometry, Nat. Commun. **6**, 6733 (2015).

[7] M. S. Wornle, P. Welter, M. Giraldo, T. Lottermoser, M. Fiebig, P. Gambardella, and C. L. Degen, Coexistence of Bloch and Neél walls in a collinear antiferromagnet, Phys. Rev. B **103**, 094426 (2021).
